# Targeting Lipopolysaccharide Transport Induces Membrane Lipid Remodeling and Sensitizes *Acinetobacter baumannii* to Colistin Treatment

**DOI:** 10.1002/advs.76198

**Published:** 2026-06-19

**Authors:** Jianya Luo, Hetianzi Zhang, Jinju Cai, Shuang Zhou, Haijie Zhang, Zhiqiang Wang, Yuan Liu

**Affiliations:** ^1^ Jiangsu Co‐innovation Center for Prevention and Control of Important Animal Infectious Diseases and Zoonoses College of Veterinary Medicine Yangzhou University Yangzhou China; ^2^ Jiangsu Key Laboratory of Zoonosis Yangzhou University Yangzhou China; ^3^ Jiangsu Interdisciplinary Center for Zoonoses and Biosafety Yangzhou University Yangzhou China

**Keywords:** Lpt system, membrane lipid reprogramming, Mla system, virtual screening

## Abstract

*Acinetobacter baumannii* is an opportunistic pathogen with increasing resistance to conventional antibiotics, necessitating novel antimicrobial strategies. The outer membrane integrity of most Gram‐negative bacteria critically depends on the lipopolysaccharide (LPS) transport (Lpt) system, which mediates LPS translocation from the inner to the outer membrane. In this study, we perform a structure‐based virtual screen against the Lpt system of *Acinetobacter* and identify a somatostatin octapeptide analogue (termed C4) as a candidate hit. Notably, C4 demonstrates modest antibacterial activity but exhibits robust synergistic activity with colistin. Integrated lipidomic and transcriptomic analyses reveal that C4 treatment induces membrane lipid remodeling, particularly a selective accumulation of phosphatidylglycerol (PG), which was associated with enhanced colistin activity. Moreover, C4 exposure significantly upregulates *mlaC* expression, a key determinant of phospholipid retrograde transport. Deletion of *mlaC* reduces C4‐induced PG enrichment and abolishes C4‐mediated potentiation of colistin, whereas *mlaC* overexpression enhances this potentiation and complementation partially restores it. In murine pneumonia and thigh infection models, the C4‐colistin combination significantly reduces bacterial burden and inflammatory cytokine levels, and attenuates histopathological damage. Our findings highlight the anti‐infective potential of targeting the Lpt system through membrane lipid remodeling and underscore C4 as a promising colistin adjuvant for combating *A. baumannii* infections.

## Graphical abstract

This study identifies C4 as a lead inhibitor of the Lpt system. Notably, C4 potentiates colistin activity by disrupting LPS transport and remodeling phospholipid homeostasis, revealing a functional interplay between the Lpt and Mla systems. These findings establish a mechanistic link between Lpt inhibition and membrane lipid remodeling, positioning Lpt–Mla axis as a promising therapeutic target against Gram‐negative infections.

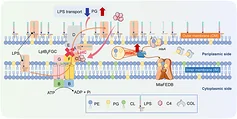

## 1 Introduction

*Acinetobacter baumannii*, an opportunistic Gram‐negative pathogen, is a leading cause of healthcare‐associated infections, including pneumonia, bloodstream infections, and wound sepsis [1]. Due to its remarkable ability to acquire and maintain resistance determinants, *A. baumannii* has become a prominent member of the ESKAPE group of pathogens, notorious for evading conventional antibiotic therapies [2]. Polymyxins, such as colistin (polymyxin E), are among the few antibiotics that retain efficacy against multidrug‐resistant (MDR) *A. baumannii*, and are often used as last‐line agents [3]. However, the emergence and dissemination of colistin resistance, driven by LPS modification [4], loss [5], or remodeling of membrane lipids [6], severely threaten their clinical utility.

Membrane integrity and lipid homeostasis are central to bacterial survival, pathogenesis, and antibiotic susceptibility [7]. In Gram‐negative bacteria, the outer membrane (OM) acts as a formidable barrier that protects cells from environmental stressors and antimicrobial agents. This asymmetric bilayer is composed of lipopolysaccharides (LPS) on the outer leaflet and phospholipids on the inner leaflet, contributing to the OM's rigidity and selective permeability [8]. Disruption of OM biogenesis or its lipid asymmetry can severely impair bacterial fitness and render cells more vulnerable to antibiotics, making OM‐targeted pathways attractive for therapeutic intervention. The lipopolysaccharide transport (Lpt) system is a highly conserved seven‐protein complex spanning from the inner membrane (IM) to the OM. It is responsible for the extraction, transport, and insertion of LPS molecules from their site of synthesis in the IM to their destination in the OM [9]. Proper LPS delivery is essential for OM stability and viability in most Gram‐negative bacteria. Notably, LPS loss has been documented in colistin‐resistant *A. baumannii* [10]. Notably, structural and biochemical studies have revealed several druggable nodes within the Lpt complex, such as LptD and LptC, making them attractive targets for novel antibacterial strategies [11, 12]. For example, a recent study demonstrated that zosurabalpin targets the LptF subunit of the inner‐membrane LptB_2_FGC complex to block endotoxin transport [13].

In parallel, the maintenance of lipid asymmetry (Mla) system is another critical membrane‐associated pathway that safeguards OM integrity. The Mla system actively transports phospholipids that have aberrantly accumulated in the outer leaflet of the OM back to the IM, thereby preserving lipid asymmetry [14]. MlaC, a periplasmic lipid‐binding protein, plays a central role by shuttling phospholipids between the IM and OM components of the Mla complex [15]. Disruption of Mla function leads to phospholipid mislocalization, membrane stress, and increased susceptibility to antibiotics [16]. The Lpt and Mla systems are traditionally studied as distinct pathways, emerging evidence suggests potential crosstalk between them in maintaining membrane homeostasis. However, their functional interplay in the context of antimicrobial susceptibility remains poorly understood. Exploring their relationship in modulating antibiotic susceptibility would guide novel therapeutic options, particularly in the development of combination strategies that compromise OM defenses and potentiate antibiotic efficacy.

In this study, we performed a high‐throughput in silico screen of a diversified chemical library against the Lpt system of Acinetobacter and identified compound C4 as a potential hit. Notably, C4 showed modest antimicrobial activity and displayed marked synergy with colistin in both checkerboard and time‐kill assays. Integrated lipidomic and transcriptomic profiling revealed that C4 treatment induced membrane lipid remodeling consistent with perturbed LPS trafficking, hallmarked by a selective accumulation of phosphatidylglycerol (PG). Genetic and biochemical analyses further demonstrated a critical role for *mlaC* upregulation in lipid remodeling and enhanced colistin‐mediated killing. Finally, murine infection models confirmed that the C4‐colistin combination provided substantial therapeutic benefit and markedly reduced tissue bacterial burdens. Collectively, our findings describe a membrane‐centered antimicrobial strategy in which Lpt perturbation‐induced lipid remodeling enhances colistin efficacy against *A. baumannii* infection.

## 2 Results

### 2.1 Virtual Screening Identifies Inhibitors of the LPS Transport Machinery

**FIGURE 1:**
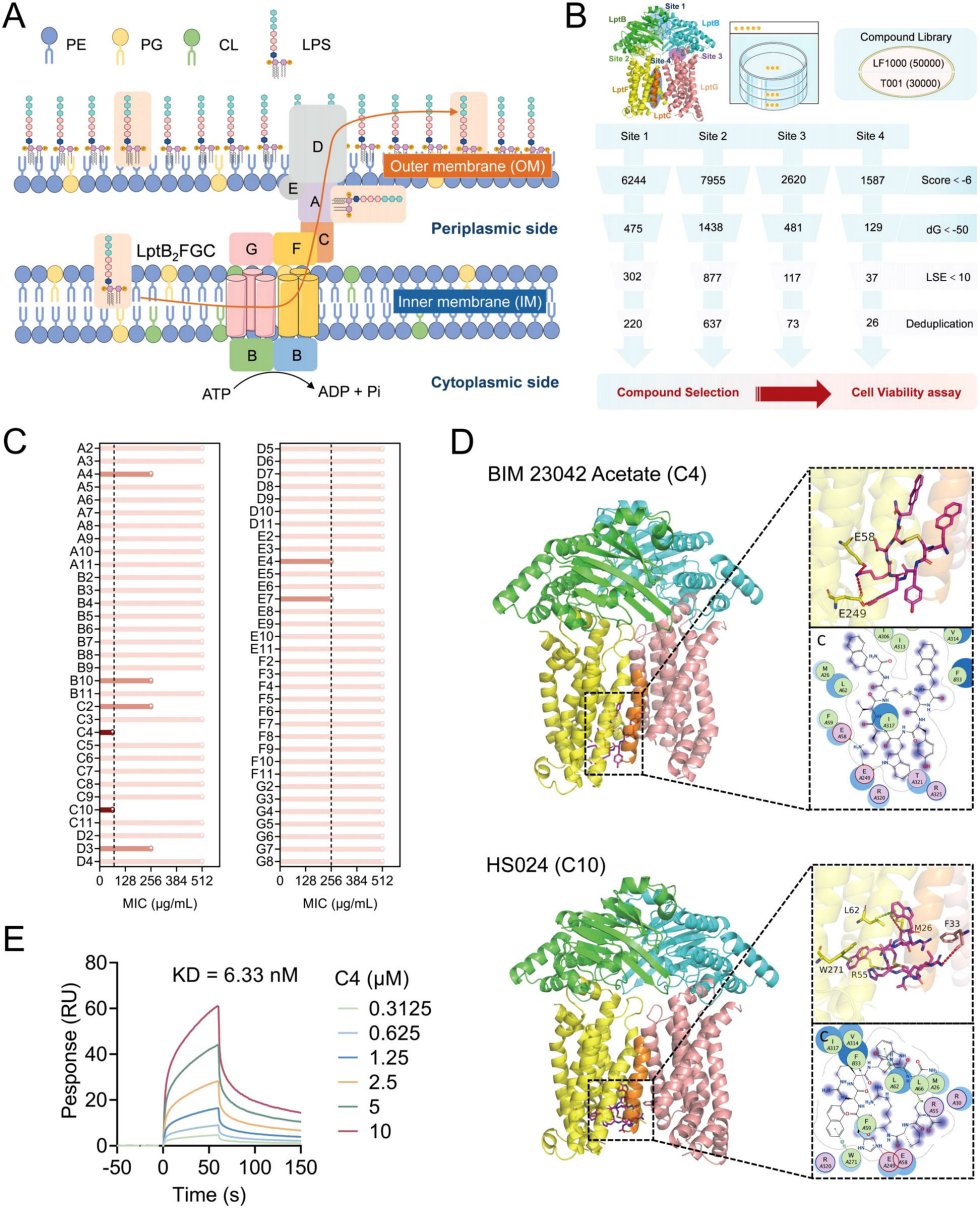
Virtual screening of compounds targeting Lpt system. (A) Schematic diagram of the Lpt heptameric complex for LPS transport. Subunits are color‐coded as follows: LptA (purple), LptB (blue and green for the two monomers), LptC (orange), LptD (gray), LptE (gray), LptF (yellow), and LptG (pink). (B) Distribution of docking scores across the four binding sites, from which compounds were selected for antimicrobial evaluation. (C) Minimum inhibitory concentrations (MICs) analysis of 67 shortlisted compounds against *A. baumannii* 19606. (D) Predicted binding poses of C4 and C10 within an LptC‐associated pocket in the LptB_2_FGC complex. (E) Surface plasmon resonance (SPR) analysis between C4 and purified LptC protein.

To maintain outer membrane biogenesis, the inner membrane components of the lipopolysaccharide transporter, LptB_2_FGC, form a subcomplex that couples ATP hydrolysis to extraction of LPS from the bilayer, passing it to the protein bridge formed by the connected β‐jellyroll domains of LptF, LptC, the soluble periplasmic protein LptA and the periplasmic portion of the integral membrane protein LptD (Figure 1A). To screen potential Lpt‐targeting compounds, we established a robust virtual‐screening pipeline combining Glide SP docking with MM‐GBSA (Molecular Mechanics Generalized Born Surface Area) rescoring (Figure 1B; Figure S1). By analyzing the structure and conformation of Lpt system, four positions were identified as possible binding regions, including the intermediate regions of LptB2 (Site 1), the intermediate regions between LptB and LptF (Site 2), the intermediate regions between LptB and LptG (Site 3), and the junctions among LptF, LptG, and LptC (Site 4). Two compound libraries, including the diversity‐based LF1000 library (50,000 molecules) and the TargetMol T001 library (30,000 molecules), were sequentially processed. After filtering by docking score and MM‐GBSA ΔG_bind, followed by structural deduplication, 220, 637, 73, and 26 non‐redundant candidates were retained for 4 potential binding sites 1–4, respectively (Figure S2). Protein–ligand interaction fingerprint (PLIF) analysis revealed recurrent hotspot residues within the LptC binding pocket (Figure S3A), while chemical‐space mapping confirmed broad scaffold diversity among the hits. We selected a subset of 67 top‐scoring compounds with the lowest MM‐GBSA ΔG values for subsequent in vitro validation. Of these, 44 have known biological activities, while 23 remain uncharacterized. These compounds are predicted to bind to four sites: Site 1 contains 5 compounds, Site 2 has 28, Site 3 includes 11, and Site 4 comprises 23.

We first assessed the antibacterial activity of these 67 hits against *A. baumannii* ATCC 19606 via minimum inhibitory concentration (MIC) assay (Table S2). Among them, two cyclic peptides, C4 (BIM 23042, a somatostatin octapeptide analogue) [17] and C10 (HS024, a receptor‐targeting cyclic peptide from prior pharmacological studies) [18] showed modest antibacterial activity, with the MIC values of 64 µg/mL, whereas the remaining 65 hits had almost no direct antibacterial effect (MIC > 128 µg/mL) (Figure 1C). Molecular docking suggested that both C4 and C10 occupy an LptC‐associated pocket at the LptB_2_FGC interface (Figure 1D), a locus not previously reported as druggable. The results indicated that C4 could bind to the key amino acid residues (e.g., E58, E249) of LptC subunit via van der Waals. Meanwhile, C10 binds to the key amino acid residues (e.g., L62, F33, M26, R55, W271) of LptC subunit through van der Waals and arene‐H. The molecular docking patterns observed between the LptB_2_FGC complex and other representative compounds was also presented (Figure S3B). To more accurately assess the binding affinity between C4 and the LptC protein, we expressed and purified recombinant LptC using a prokaryotic expression system (Figure S4). Subsequently, we employed surface plasmon resonance (SPR) analysis to evaluate the binding affinity between C4 and LptC. The results demonstrated that C4 directly interacts with LptC, exhibiting a strong binding affinity characterized by an equilibrium dissociation constant (KD) of 6.3 × 10^−6^ M (Figure 1E).

To substantiate the involvement of LptC in C4 activity, we isolated a C4‐resistant mutant exhibiting a twofold increase in MIC (designated C4‐Mutant; Figure S5A). Sanger sequencing identified a single nucleotide substitution in *lptC* (Figure S5B), supporting an association between *lptC* variation and reduced C4 susceptibility. To validate this finding, we constructed CRISPRi‐mediated *lptC* knockdown and overexpression strains. RT–qPCR confirmed the respective reduction and elevation of *lptC* expression (Figure S6A). Consistent with a role in C4 susceptibility, the MIC decreased from 64 to 32 µg/mL in the knockdown strain, whereas overexpression increased the MIC from 64 to 128 µg/mL (Figure S6B). Together, these data nominate LptC as a potential antibacterial target and underscore the therapeutic potential of cyclic‐peptidomimetic inhibitors against the LPS transport machinery.

### 2.2 C4 Enhances the Antibacterial Activity of Colistin Against *A. baumannii*

**FIGURE 2:**
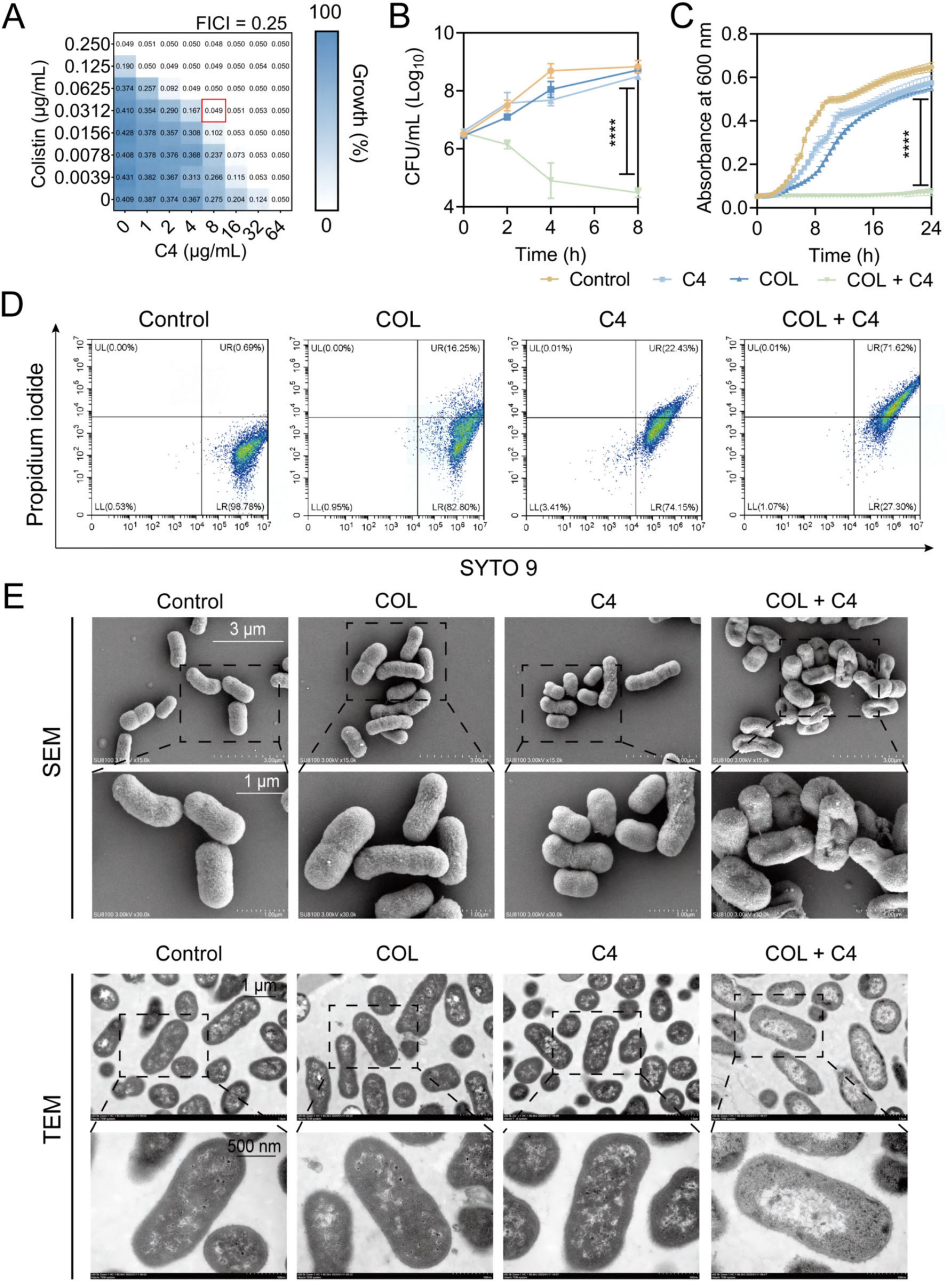
C4 potentiates colistin activity against *A. baumannii*. (A) Synergistic inhibition of bacterial growth by C4 and colistin, assessed by checkerboard assay and fractional inhibitory concentration index (FICI). (B) Time‐kill curves of C4‐colistin combination against *A. baumannii* 19606. (C) Growth curves of *A. baumannii* 19606 treated with C4, colistin, or both over 24 h. (D) Flow cytometry analysis of live/dead bacteria following 6 h treatments. (E) SEM/TEM imaging of bacterial ultrastructure after 8 h of treatment. Data are shown as mean ± SD. *p* value was determined by two‐way ANOVA (B, C). ****p < 0.0001.

Given the pivotal role of the Lpt system in maintaining membrane homeostasis, we hypothesized that pharmacological inhibition of this pathway might potentiate colistin activity against *A. baumannii*. To test this hypothesis, we assessed the synergistic activity between C4 and colistin against *A. baumannii* 19606 through checkerboard assays. The results indicated that C4 exhibited a pronounced synergistic interaction with colistin against *A. baumannii* 19606, yielding a FICI of 0.25 (Figure 2A). Furthermore, we examined the C4–colistin combination against additional Enterobacteriaceae (*Klebsiella pneumoniae*, *Escherichia coli* and *Salmonella* *spp*.) and *Pseudomonas aeruginosa*, which exhibited synergistic effects (Figure S7). Time‐kill curves and growth kinetics further demonstrated that neither sub‐MICs of C4 nor colistin alone exerted substantial bactericidal or bacteriostatic activity, whereas their combination reduced viability counts by > 4 log_10_ within 8 h (Figure 2B,C). Flow‐cytometric quantification of SYTO9/PI‐stained cells corroborated these findings, revealing a marked shift from viable to non‐viable populations under combination therapy (Figure 2D).

Furthermore, we assessed their synergistic effect via ultrastructural observation. Scanning electron microscopy (SEM) showed intact, rod‐shaped cells in untreated and monotherapy controls, whereas the C4‐colistin regimen induced extensive surface collapse and membrane blebbing. Transmission electron microscopy (TEM) revealed intact cell envelopes in control groups, but combination treatment provoked severe disruption of both peptidoglycan and outer‐membrane layers (Figure 2E). Consistently, confocal laser scanning microscopy (CLSM) visualized a pronounced reduction in green (live) fluorescence and concomitant increase in red (dead) fluorescence, underscoring the superior bactericidal efficacy of the C4‐colistin combination (Figure S8). Collectively, these data demonstrate that C4 perturbs membrane homeostasis and enhances colistin activity against *A. baumannii*.

### 2.3 C4 Perturbs Phospholipid Metabolism

**FIGURE 3:**
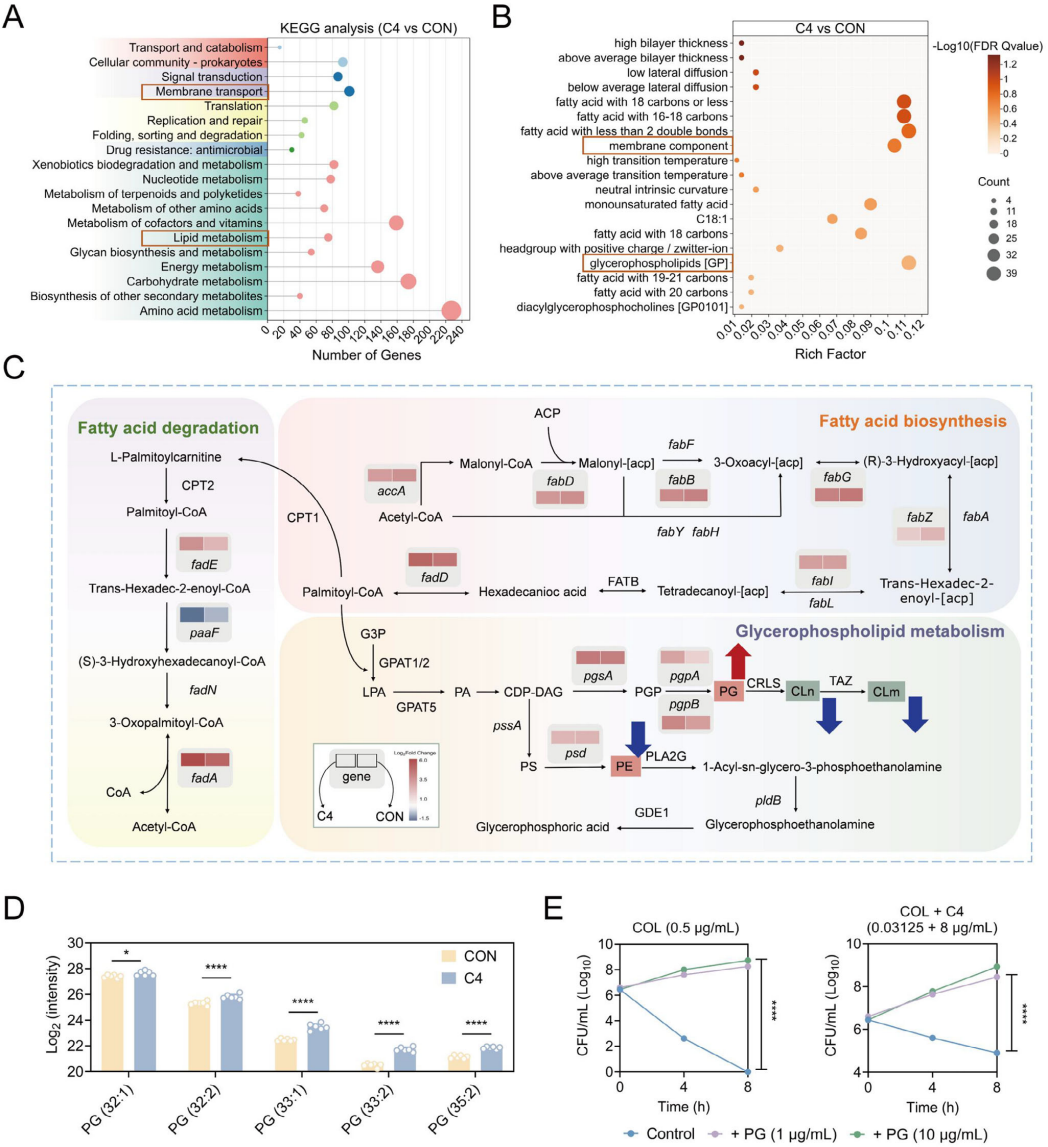
C4 remodels phospholipid metabolism. (A) KEGG pathway enrichment analysis of DEGs. (B) Pathway mapping using MetaboAnalyst emphasized glycerophospholipid and membrane‐related enrichment. (C) Transcriptional and metabolic changes in fatty acid degradation, biosynthesis, and glycerophospholipid metabolism upon C4 treatment. (D) Quantitative comparison of PG between C4 and control groups. (E) The effects of exogenous PG supplementation on the efficacy of colistin alone and on the synergy between C4 and colistin. Data are shown as mean ± SD. *p* value was determined by two‐way ANOVA (D, E). *p < 0.05, ****p < 0.0001.

To elucidate the specific modes of action of candidate compound C4, we first performed transcriptomic analysis of *A. baumannii* 19606 cells treated with or without C4. RNA‐seq identified 440 differentially expressed genes (DEGs, |log_2_FC| ≥ 1, FDR < 0.05), including 327 up‐regulated and 113 down‐regulated (Figure S9A). KEGG enrichment analysis underscored significant perturbations in membrane transport and glycerophospholipid metabolism (Figure 3A and Figure S9B). We next sought to verify the roles of phospholipid metabolism perturbation by C4 in its potentiation to colistin. First, we performed RT‐qPCR analysis for representative DEGs to validate transcriptomic trends. Consistently, the expression of *pgpA* and *fadE* was significantly upregulated, while *paaF*, *dhaT*, and *purK* were markedly downregulated (Figure S9C), indicating that C4 exposure excessively activated the PG biosynthetic pathway while repressing genes involved in fatty acid degradation. Transcriptomic analysis also revealed upregulation of genes involved in membrane transport, including those from two‐component systems, ABC transporters, and bacterial secretion systems. RT‐qPCR confirmed significant upregulation of *wza*, *rstA*, *lolE*, *mlaC*, *sbp*, *scuA*, and *vgrG* (Figure S9D), suggesting a robust activation of the ABC transporter network in response to C4.

According to the findings of transcriptomic study, we next conducted lipidomic profiling to reveal the changes of phospholipid metabolism under C4 treatment. Untargeted lipidomic showed a pronounced metabolic shift, with principal component analysis (PCA), partial least squares‐discriminate analysis (PLS‐DA) demonstrating a clear separation between control and C4‐treated samples (Figure S10A). We identified 489 significantly altered lipid species (*p* < 0.05, |FC| ≥ 2), comprising 176 up‐ and 313 down‐regulated metabolites (Figures S10B and S11). Among these, cardiolipin (CL), phosphatidylethanolamine (PE), and phosphatidylglycerol (PG) represented the most responsive lipid families (Figure S10C). KEGG pathway enrichment analysis showed notable enrichment in glycerophospholipid metabolism and fatty acid degradation pathways (Figure S10D). Additionally, functional enrichment analysis of lipid categories revealed significant perturbations in membrane‐associated components and glycerophospholipids (Figure 3B).

To elucidate the correlation between transcriptomic and lipidomic alterations, we employed iPath3.0 [19] to construct integrated metabolic maps for glycerophospholipid metabolism, fatty acid biosynthesis, and degradation pathways (Figure S12), demonstrating the tightly interconnected nature of these pathways. Specifically, C4 resulted in downregulation of the glycerophospholipid metabolism and fatty acid biosynthesis pathways, accompanied by upregulation of the fatty acid degradation pathway (Figure 3C), indicating that C4 remodels membrane phospholipids. Notably, lipidomic results showed that C4 treatment markedly elevated PG, including PG (32:1), PG (32:2), PG (33:1), PG (33:2) and PG (35:2), while reducing CL and PE abundance (Figure 3D and Figure S10E). Based on this finding, we further assessed the impact of PG supplementation on bacterial susceptibility to colistin alone and in combination with C4. Notably, exogenous PG completely abrogated colistin‐mediated killing of *A. baumannii* 19606 and markedly attenuated the synergistic activity between C4 and colistin (Figure 3E). These results reveal a functional contribution of phospholipid remodeling, specifically PG redistribution, to the C4‐mediated potentiation of colistin.

### 2.4 *mlaC* is Required for C4–Colistin Synergy

**FIGURE 4:**
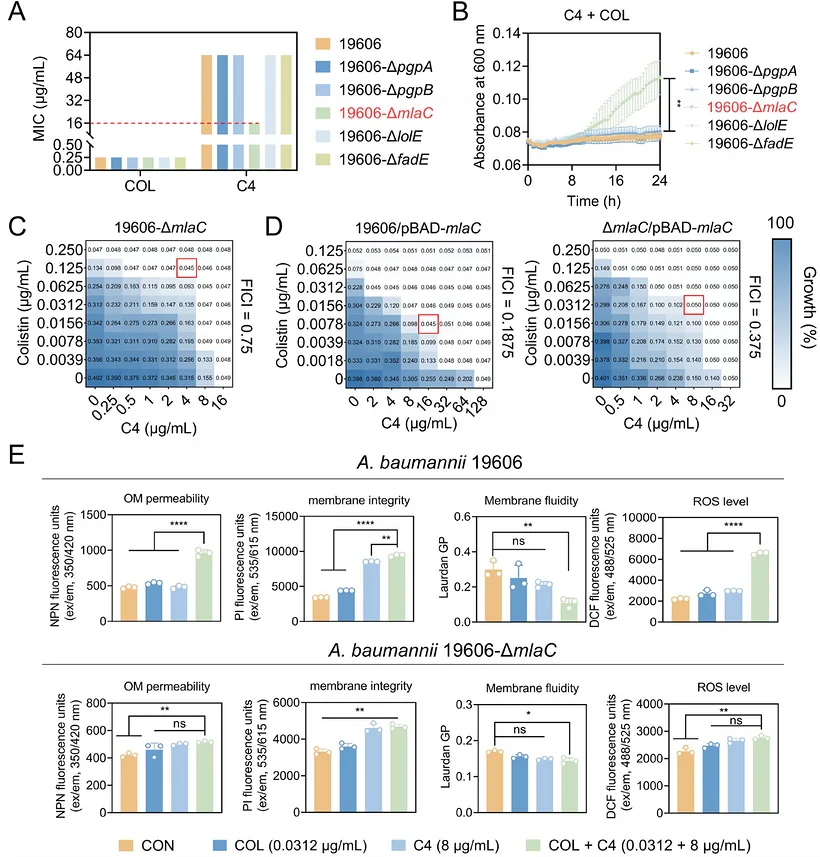
*mlaC* is required for C4‐mediated colistin sensitization. (A) MIC analysis for C4 and colistin against WT and five gene‐deletion strains. (B) Growth curves of WT and gene‐deletion strains under C4 plus COL treatment. (C and D) Checkerboard assay of C4 and colistin in the ∆*mlaC* strain, the *mlaC* overexpression and complementation strains. (E) Membrane integrity, permeability, fluidity, and ROS levels in WT and ∆*mlaC* after exposure to C4, colistin alone or their combination. Data are shown as mean ± SD. *p* value was determined by two‐way ANOVA (B) or one‐way ANOVA (E). *p < 0.05, **p < 0.01, ***p < 0.001, ****p < 0.0001. ns, not significant.

To decipher the specific pathways that mediates the synergistic effect between C4 and colistin, we tried to knockout the key upregulated genes involved in membrane transport and phospholipid metabolism upon C4 treatment. Using the pRE112 suicide vector system, we successfully constructed five gene deletion mutants of *A. baumannii* 19606, including Δ*pgpA* and Δ*pgpB* (glycerophospholipid metabolism), Δ*mlaC* and Δ*lolE* (ABC transport), and Δ*fadE* (fatty acid degradation). MIC assays revealed that only the Δ*mlaC* strain exhibited increased susceptibility to C4, with the MIC value decreasing from 64 to 16 µg/mL (Figure 4A). Interestingly, bacterial growth analysis showed that only the Δ*mlaC* mutant was able to survive under C4 and colistin co‐treatment conditions (Figure 4B). Consistently, checkerboard assays showed that the synergistic effect between C4 and colistin was abolished in the Δ*mlaC* strains with a FICI of 0.75 (Figure 4C). To further validate this genetically, we constructed an *mlaC* overexpression strain (19606/pBAD‐*mlaC*) and a complementation strain (Δ*mlaC*/pBAD‐*mlaC*). As a result, we found that overexpression of *mlaC* increased susceptibility to colistin (twofold MIC reduction) but decreased susceptibility to C4 (MIC increased to 128 µg/mL) (Figure S13). Meanwhile, checkerboard assays revealed markedly enhanced synergy against 19606/pBAD‐*mlaC* (FICI = 0.1875) compared with WT, whereas complementation partially restored synergy in the Δ*mlaC* background (FICI = 0.375) (Figure 4D). These results suggested that *mlaC* was essential for the synergistic effect between C4 and colistin.

To elucidate how *mlaC* loss alters cellular responses to C4, we compared transcriptomic profiles of WT and Δ*mlaC* strains following C4 exposure. Genes associated with two‐component systems, bacterial secretion systems, and ABC transporters were significantly upregulated in the Δ*mlaC* mutant (Figure S14), suggesting that *mlaC* deletion disrupts membrane transport homeostasis and triggers compensatory stress responses. We next examined whether *mlaC* deletion alters membrane homeostasis under C4 and colistin stress. Using NPN uptake (outer membrane permeability), PI staining (membrane integrity), and Laurdan GP measurements (membrane fluidity), we found that the Δ*mlaC* mutant maintained greater membrane stability than WT cells upon treatment. Consistently, intracellular ROS accumulation was significantly attenuated in Δ*mlaC* (Figure 4E), indicating that *mlaC* deletion confers protection against C4‐ and colistin‐induced membrane damage.

**FIGURE 5:**
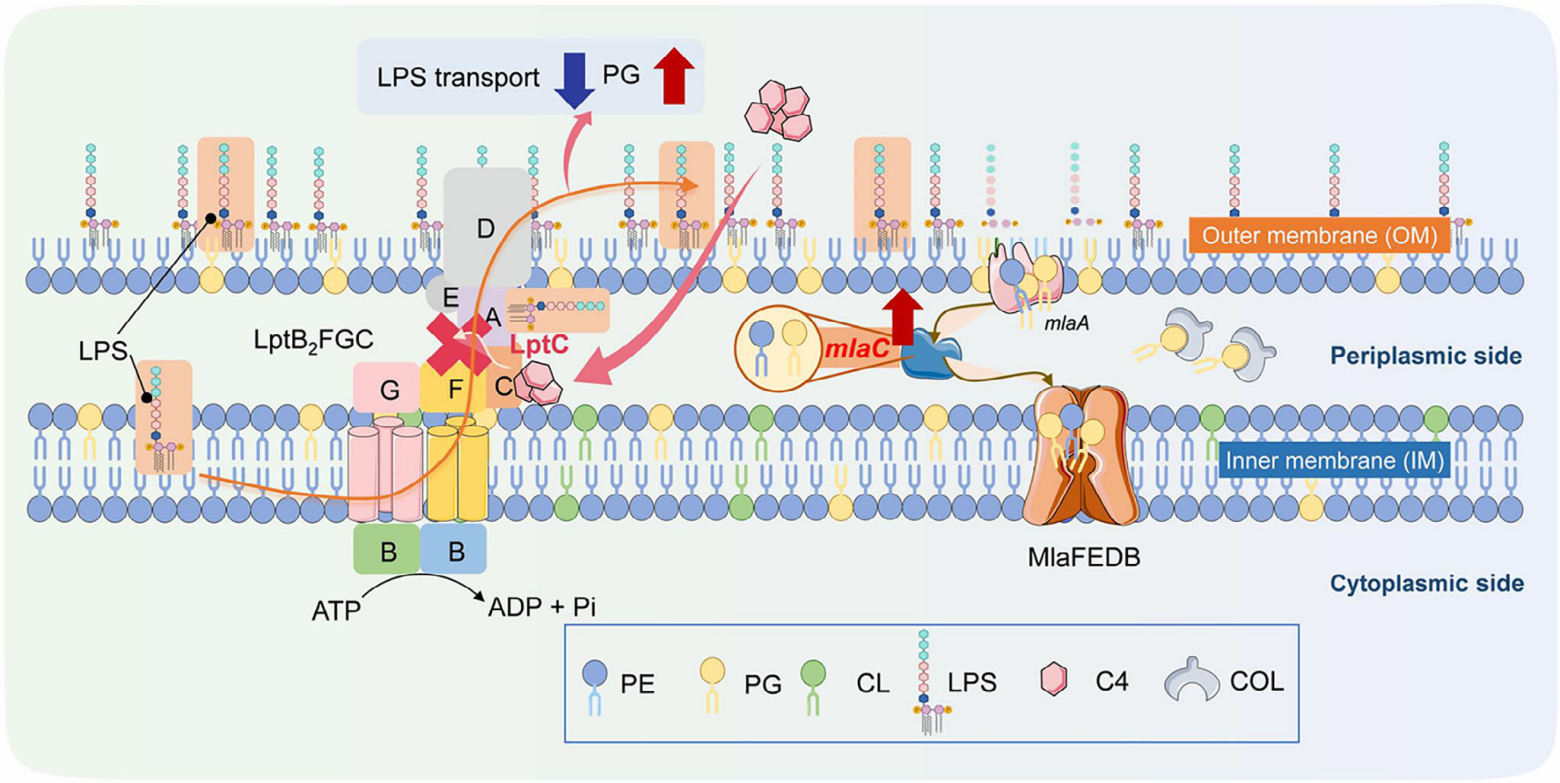
Synergistic mechanisms between C4 and colistin against *A. baumannii*. C4 perturbs LptC‐associated LPS trafficking, potentially reducing LPS delivery to the outer membrane while causing envelope lipid stress, thereby inducing MlaC‐dependent phospholipid remodeling. This remodeling, particularly involving PG redistribution, contributes functionally to a membrane state that is more susceptible to colistin.

Collectively, these data support a model in which C4 disrupts LptC‐dependent LPS transport, leading to compromised envelope asymmetry. This defect is coupled to extensive phospholipid remodeling and concomitant activation of the Mla pathway. We further show that *mlaC*‐dependent lipid redistribution, specifically of PG, is required for C4‐mediated colistin potentiation, indicating that C4–colistin synergy arises from an integrated Lpt–Mla remodeling axis (Figure 5).

### 2.5 C4 Treatment Exhibits Favorable Safety Profile

**FIGURE 6:**
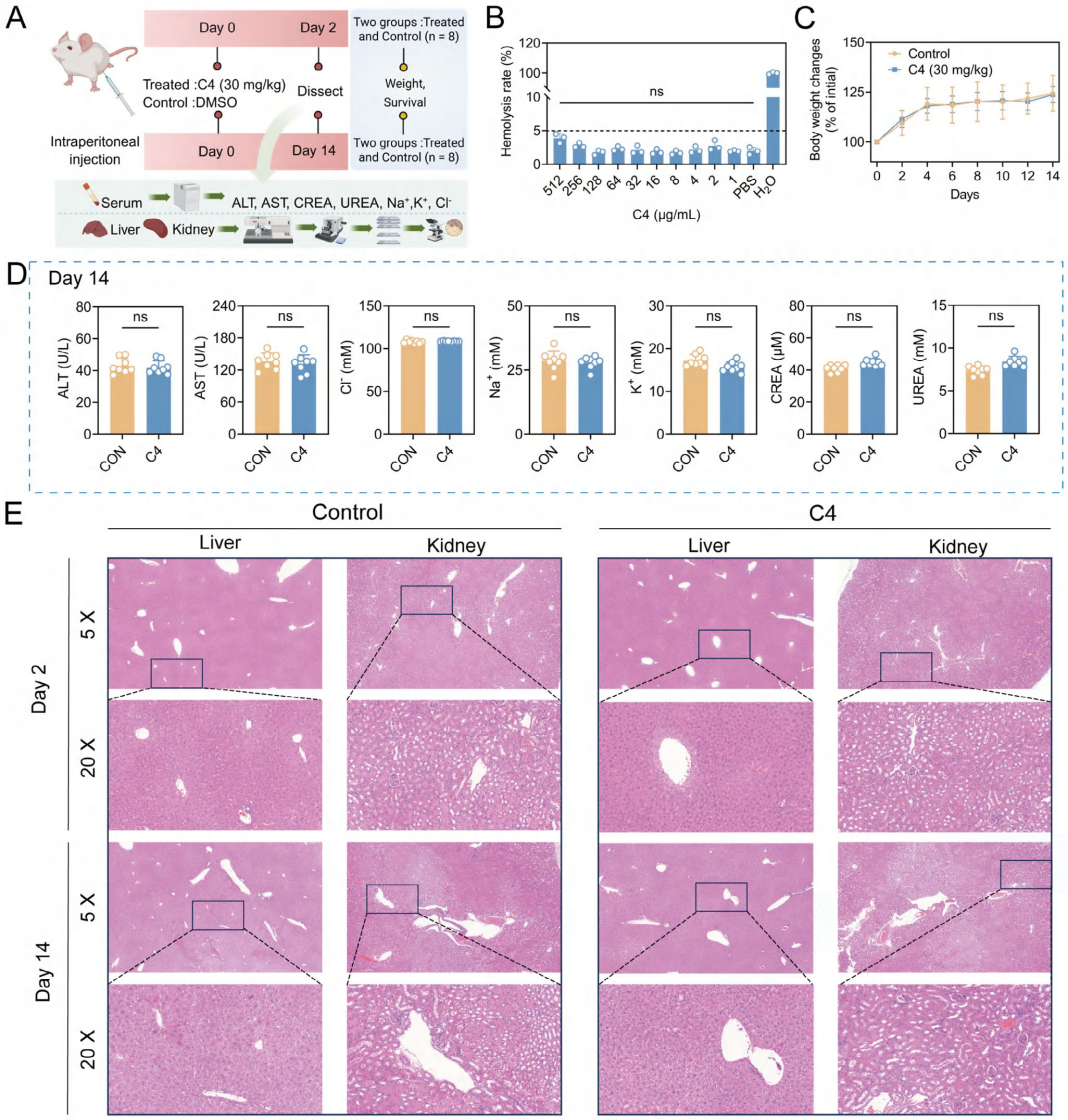
C4 demonstrates favorable in vivo safety in mice. (A) Experimental protocols. BALB/c mice received a single intraperitoneal injection of vehicle or C4 (30 mg/kg) and were monitored for 14 days. (B) Hemolytic activity of C4 against RBCs. (C) Body weight of mice over the treatment period. (D) Serum biochemistry anlsysis on Day 14. (E) H&E staining of mice liver and kidney. Data are shown as mean ± SD. *p* value was determined by one‐way ANOVA (B) or unpaired *t*‐test (D). ****p < 0.0001. ns, not significant.

To comprehensively evaluate the in vivo safety profile of compound C4, we conducted a series of experiments using 7‐week‐old male BALB/c mice (Figure 6A). Mice were intraperitoneally administered a single dose of 30 mg/kg C4, and sacrificed at two distinct time points, 2‐ and 14‐days post‐treatment, via cervical dislocation. Blood samples were collected to assess key indicators of hepatic and renal function, as well as serum electrolyte levels. Concurrently, liver and kidney tissues were harvested for histopathological examination. Meanwhile, the hemolytic activity of C4 against mammalian red blood cells (RBCs) was evaluated to assess potential cytotoxicity. Notably, even at a high concentration of 512 µg/mL, C4 induced minimal hemolysis (Figure 6B), indicating excellent biocompatibility with erythrocytes.

During the entire observation period, no mortality or abnormal behavioral changes were observed in the mice treated with C4. Furthermore, C4 administration had no significant impact on body weight, serum biochemical indices, or overall physiological condition (Figure 6C,D; Figure S15). Liver enzyme levels, including alanine transaminase (ALT) and aspartate transaminase (AST), remained within normal ranges, thereby confirming the absence of hepatotoxicity. Similarly, normal concentrations of blood urea nitrogen (BUN) and creatinine (CREA) ruled out nephrotoxic effects. Additionally, electrolyte levels, including sodium (Na^+^), potassium (K^+^), and chloride (Cl^−^), remained stable, suggesting that C4 treatment did not disrupt systemic ion homeostasis.

Histological analysis using hematoxylin and eosin (H&E) staining revealed no observable pathological lesions or tissue damage in either the liver or kidneys of C4‐treated mice when compared to the vehicle‐treated control group (Figure 6E). Collectively, these results demonstrate that C4 is a well‐tolerated and non‐toxic compound, with excellent in vivo safety characteristics, thereby supporting its further evaluation as a potential colistin adjuvant.

### 2.6 C4 Potentiates the In Vivo Efficacy of Colistin

**FIGURE 7:**
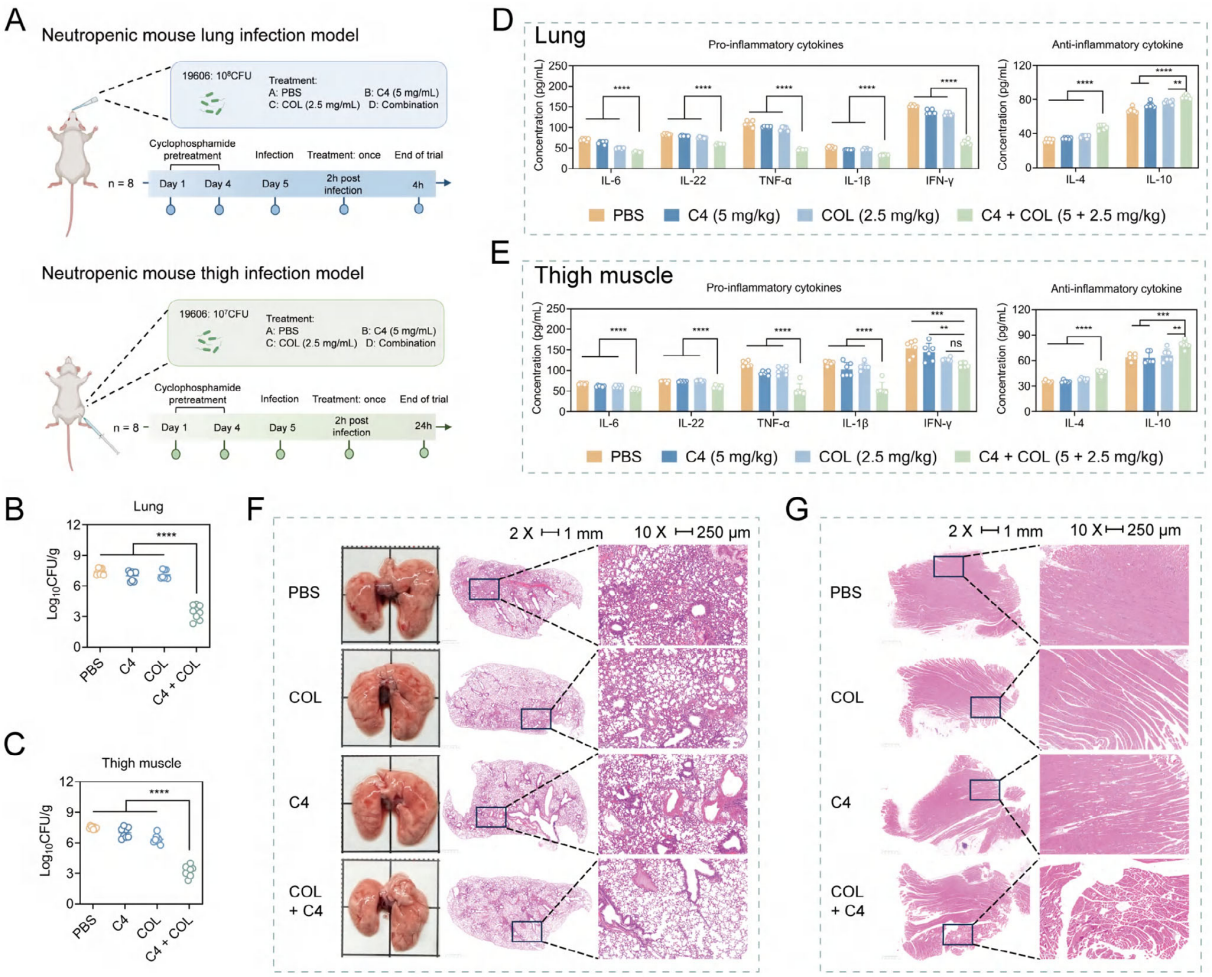
C4 and colistin show synergistic therapeutic efficacy in vivo. (A) Experimental design for neutropenic mouse models of lung and thigh infections (*n* = 8 per group). (B, C) Bacterial burdens in mice lung (B) and thigh muscle (C) under C4 plus colistin therapy. (D, E) Cytokine profiling in mice lung (D) and thigh muscle (E) following combination treatment. (F, G) H&E staining of mice lung (F) and thigh muscle (G) under different treatments. Data are shown as mean ± SD. *p* value was determined by one‐way ANOVA (B, C) or two‐way ANOVA (D, E). **p < 0.01, ***p < 0.001, ****p < 0.0001. ns, not significant.

Having demonstrated the potentiating effect of C4 on colistin in vitro, we next evaluated its therapeutic benefit in vivo using two neutropenic mouse infection models (Figure 7A). In the pneumonia model, combination therapy significantly reduced bacterial burden in the lungs (Figure 7B). In the thigh infection model, bacterial loads were also markedly lower in the combination group than in either single‐treatment group (Figure 7C). Moreover, compared with monotherapy or untreated controls, cytokine analysis showed decreased pro‐inflammatory and partially normalized anti‐inflammatory cytokine levels (Figure 7D,E). Histopathological evaluation further corroborated these findings: lung sections from infected and monotherapy‐treated mice displayed severe alveolar collapse and perivascular congestion, whereas the combination group exhibited preserved alveolar architecture and reduced tissue injury (Figure 7F). Similarly, thigh muscle sections from mice receiving the combination therapy showed substantially less tissue destruction, confirming the superior therapeutic efficacy of the C4–colistin regimen (Figure 7G).

## 3 Discussion

*Acinetobacter baumannii*, a Gram‐negative opportunistic pathogen, causes severe nosocomial infections worldwide. The rapid spread of MDR strains, which demonstrate resistance to both first‐line carbapenems and last‐resort agents like colistin, has rendered conventional therapies increasingly ineffective, leading to elevated mortality rates [20, 21]. In light of this critical public health threat, World Health Organization (WHO) and the U.S. Centers for Disease Control and Prevention (CDC) have classified MDR *A. baumannii* as an urgent threat, emphasizing the urgent need for innovative treatment strategies [22]. Consequently, given the stagnancy of traditional antibiotic discovery, the search for adjuvants that restore existing antibiotics and the exploration of novel targets have become promising strategies to combat difficult‐to‐treat infections [23, 24]. In this study, we employed a structure‐based virtual screening approach to identify Lpt‐targeting compounds against *A. baumannii*. This screen yielded a somatostatin octapeptide analogue, designated C4, as a putative LptC‐associated hit. Functionally, C4 exhibited modest direct antibacterial activity yet markedly potentiated colistin, providing a potential therapeutic strategy to combat *A. baumannii* infections.

The OM of Gram‐negative bacteria is a highly organized asymmetric bilayer whose outer leaflet, dominated by LPS, serves as a formidable barrier to antimicrobial agents [12]. Consequently, OM biogenesis pathways, including LPS transport and β‐barrel protein assembly, have emerged as compelling antibacterial targets [25]. BamA, the essential core of the β‐barrel assembly machinery (BAM), catalyzes outer membrane protein insertion; its inhibition disrupts membrane integrity and ultimately leads to bacterial death [26]. Similarly, the LPS transport (Lpt) system is indispensable for OM stability in most Gram‐negative bacteria, forming a trans‐periplasmic bridge to deliver LPS to the outer leaflet [27]. This machinery presents a dual vulnerability, as its disruption not only impairs membrane assembly but also sensitizes bacteria to existing antibiotics [28]. Tethered macrocyclic peptides (MCPs) recently validated this paradigm, trapping the Lpt complex in an LPS‐bound state to achieve potent bactericidal activity against *A. baumannii* [13]. Our work extends this concept, demonstrating that LptC targeting potentiates conventional antibiotics beyond simple biogenesis impairment. These multifaceted benefits position the Lpt pathway as a uniquely promising target for recalcitrant Gram‐negative infections. Membrane lipid composition further modulates antibiotic susceptibility. The negative surface charge of PG facilitates colistin binding, promoting membrane disruption [29, 30, 31]. Consistently, our findings reveal that C4‐induced PG remodeling functionally underpins this enhanced colistin activity. Notably, while C4‐mediated inhibition of the Lpt system can reduce surface‐exposed outer‐membrane LPS, its effect on intracellular LPS levels remains unclear, as mislocalized LPS maybe degraded in certain Gram‐negative bacteria, including *E. coli* [32].

The maintenance of lipid asymmetry (Mla) system preserves OM lipid asymmetry by recovering phospholipids that are misplaced [33]. Structurally, the Mla system consists of the OM complex MlaA–OmpC/F, the periplasmic shuttle protein MlaC, and the IM ABC transporter MlaBDEF [34]. The main role of the Mla system is to extract aberrant phospholipids (predominantly PG) from the OM outer leaflet and shuttle them to the inner membrane through MlaC, thus sustaining OM lipid balance [15, 35]. Deletion of *mlaF* [14] or *mlaC* impairs bacterial tolerance to membrane stress and antibiotics, indicating that the intact Mla system is required for bacterial survival and virulence. Interestingly, our work demonstrated that inhibiting LptC disrupts outer membrane lipid asymmetry, which activates compensatory mechanisms mediated by the Mla system [16]. Specifically, we found that C4, by targeting LPS transport, triggers membrane stress and subsequently drives the upregulation of *mlaC* expression to restore OM integrity. This finding is consistent with prior reports that under selective pressure from membrane‐active peptides such as arenicin‐350, bacteria often acquire mutations or alter *mlaC* expression [36]. These observations further underscore the Mla pathway as a pivotal defense hub against outer membrane disruption.

Functionally, the Lpt and Mla systems act in concert to maintain lipid homeostasis, with the Lpt pathway exporting LPS to the OM and the Mla pathway removing excess phospholipids [37]. This coordination implies that impaired LPS transport creates a dependency on Mla‐mediated retrograde phospholipid trafficking, which is indispensable for bacterial survival [38]. Critically, without this compensatory mechanism, bacteria cannot establish a membrane environment contributing to boost colistin activity. Directly supporting this finding, we found that *mlaC*‐deficient mutants are unable to re‐accumulate PG in the inner membrane, thereby abolishing the synergistic killing between Lpt inhibitors and colistin. Consistent with this crucial role of MlaC, we further demonstrated that overexpression of the *mlaC* significantly enhanced the potentiation effect of C4 to colistin. Moreover, complementation of *mlaC* appropriately restored the ability of C4 to enhance colistin activity.

Compound C4, a somatostatin octapeptide analogue originally developed as a selective neuromedin B receptor antagonist with well‐characterized pharmacological properties [17]. Repurposing C4 as an anti‐infective agent with multifaced functions represents an innovative therapeutic strategy. The present findings support direct C4–LptC interaction and are consistent with disruption of Lpt‐dependent LPS transport. Notably, the C4–colistin combination markedly enhanced bactericidal activity both in vitro and in vivo, while exhibiting negligible toxicity. Clinically approved somatostatin analogues, including octreotide [39] and lanreotide [40], have been administered for decades with excellent safety and tolerability profiles, suggesting that C4 may possess favorable pharmacokinetic properties and safety potential. Future optimization of C4 will focus on enhancing membrane penetration and stability (e.g., via cyclization [41] or incorporation of non‐natural amino acids [42]), strengthening LptC affinity, and improving delivery routes to broaden antibacterial spectrum and efficacy.

In summary, our study identifies multiple Lpt system inhibitors, with C4 as the lead compound, and defines their anti‐infective mechanisms. Mechanistically, C4 potentiates colistin activity by disrupting LPS transport and remodeling phospholipid homeostasis, revealing a previously unrecognized functional interplay between the Lpt and Mla systems. This interplay exposes a vulnerability in bacterial membrane homeostasis that can be therapeutically exploited through co‐administration of Lpt inhibitors with existing antibiotics to resensitize drug‐resistant Gram‐negative pathogens. Collectively, our findings establish a mechanistic link between Lpt inhibition and membrane lipid remodeling, positioning the Lpt–Mla axis as a promising therapeutic target against multidrug‐resistant Gram‐negative infections.

## 4 Materials and Methods

### 4.1 Protein Preparation

The receptor model was prepared from the cryo‐EM structure of the *A. baylyi* LptB_2_FGC complex (PDB ID: 8FRP; resolution: 3.80 Å), which served as a surrogate template for the *A. baumannii* Lpt system in docking analyses. All antibacterial, genetic, and biochemical validation experiments were performed in *A. baumannii* strains. Protein structures were processed using the Protein Preparation Wizard module in the Schrödinger software suite [43], which included optimization of bond orders, addition of hydrogen atoms, assignment of disulfide bonds, and determination of protonation states using the PROPKA algorithm at pH 7.0. Energy minimization was performed with the OPLS4 force field to resolve atomic clashes and optimize side‐chain conformations, with heavy‐atom root‐mean‐square deviation (RMSD) converged to 0.3 Å to ensure structural stability [44]. The optimized structure was saved as a PDB file and used as the receptor model for subsequent virtual screening.

### 4.2 Ligand Preparation

The ligand dataset comprised two compound libraries: LF1000, a known compound library containing 50,000 molecules, and T001, a novel compound library containing 30,000 molecules. Ligand preparation was conducted using the LigPrep module in Schrödinger (LigPrep, Schrödinger, LLC, New York, NY, 2021). Protonation states were assigned, salts were removed, and tautomers were generated using the Epik method at pH 7.0 ± 2.0, with preservation of original stereochemistry. To ensure comprehensive conformational sampling during virtual screening, up to 32 low‐energy conformers were generated per compound. The processed libraries were saved as ligand input files for downstream docking procedures.

### 4.3 Virtual Screening

Prepared receptor and ligand files were imported into the Glide docking module. Virtual screening was performed using Glide‐SP (standard precision) docking followed by MMGBSA binding free energy calculation [45]. Flexible ligand docking was applied with default parameters, and energy optimization was conducted after docking. The top 3 conformations from SP docking were retained, and the top 10% of scored conformers were subjected to MMGBSA calculation. Compounds were filtered using thresholds of docking score < ‐6 kcal/mol, binding free energy dG < ‐50 kcal/mol, and ligand strain energy < 10 kcal/mol. Candidates were further clustered and deduplicated based on protein–ligand interactions and a structural similarity cutoff of 70%. Protein‐ligand interaction fingerprinting (PLIF), structural diversity assessments, and binding mode analyses were conducted to characterize potential binding interactions.

### 4.4 Bacterial Strains and Chemical Reagents

All bacterial strains used in this study are listed in Table S1. The mutant of *A. baumannii* 19606 was constructed via homologous recombination using the suicide plasmid pRE112 and verified by PCR as previously described [46]. Unless otherwise specified, bacterial cultures were grown at 37°C in cation‐adjusted‐Mueller‐Hinton broth (CAMHB), Luria‐Bertani broth (LB; Qingdao Hope Bio‐Technology), or on LB agar (LBA) plates. Antibiotics and other chemical compounds were purchased from TargetMol (Shanghai, China). Arabinose refers specifically to L‐arabinose.

### 4.5 Broth Microdilution Assay

The minimum inhibitory concentrations (MICs) of antibiotics and non‐antibiotics were determined using the standard broth microdilution method in accordance with CLSI 2021 guidelines. Briefly, bacteria in exponential phase were diluted 1:1000 in CAMHB, and 5 × 10^5^ CFU/mL bacterial suspensions were mixed with serially diluted compounds in sterile 96‐well plates. After incubation at 37°C for 16–18 h, the MIC was defined as the lowest concentration that completely inhibited visible growth [47].

### 4.6 Checkerboard Analysis

Synergistic activity was assessed using a checkerboard assay. Twofold serial dilutions were prepared in an 8 × 8 matrix. After incubation with 5 × 10^5^ CFU/mL bacterial suspensions for 18 h at 37°C, OD_600_ was measured using a microplate reader. The fractional inhibitory concentration index (FICI) was calculated as: FICI = FICa + FICb = MICab/MICa + MICba/MICb, where a FICI ≤ 0.5 was interpreted as synergistic [48].

### 4.7 Time‐Dependent Killing Curve

An overnight culture of *A. baumannii* 19606 was diluted 1:100 in fresh CAMHB and incubated for 4 h at 37°C with shaking (200 rpm). Cultures were then treated with C4 (8 µg/mL), colistin (0.03125 µg/mL), or their combination for 8 h. Samples were collected at 0, 2, 4, and 8 h, serially diluted, and plated for CFU enumeration. PBS in MHB was used as a negative control. Experiments were performed in triplicate [49].

### 4.8 Growth Curves Measurement

Overnight cultures were diluted 1:1000 in 1 mL fresh CAMHB and subjected to various treatments. Cultures (200 µL per well) were grown in UV‐sterilized 96‐well plates with 50 µL mineral oil added to minimize evaporation. OD_600_ was continuously measured using an Infinite E Plex microplate reader (Tecan).

### 4.9 Flow Cytometry Analysis

Overnight cultures were diluted 1:100 and grown for 4 h, centrifuged, washed, and resuspended in PBS to an OD_600_ of 0.5. Bacteria (10^6^ CFU/mL) were treated with C4 (8 µg/mL), colistin (0.03125 µg/mL), or both for 6 h. Cells were stained with 3 µL of PI (5 mM) and 3 µL of SYTO 9 (0.835 mM) for 15 min in the dark at room temperature. Fluorescence was detected using a CytExpert Flow Cytometer (Beckman, USA), and 100,000 ungated events were analyzed using CytExpert 2.0 [50].

### 4.10 Scanning Electron Microscopy (SEM)

SEM was conducted as previously described [51]. In brief, exponential‐phase bacterial cultures (10^6^ CFU/mL) were incubated with C4 and/or colistin for 8 h at 37°C. Bacterial suspensions were centrifuged (5000 × g, 5 min) and gently washed twice with PBS. The bacterial pellets were then fixed with 2.5% glutaraldehyde (Solarbio, Beijing, China) for 24 h at 4°C. Subsequently, bacteria were dehydrated through a series of ethanol gradients (40%, 50%, 60%, 70%, 80%, 90%, and 100%; 10 min each). After gold sputtering, bacterial morphology was detected with GeminiSEM 300 (ZEISS, Germany).

### 4.11 Transmission Electron Microscopy (TEM)

TEM was performed as described previously [52]. Briefly, exponential‐phase bacterial cultures (10^6^ CFU/mL) were incubated with the drugs for 8 h at 37°C. Bacterial suspensions were then centrifuged and resuspended in 1 mL of a fixative solution composed of 2.5% glutaraldehyde and 5% formaldehyde. Fixed bacteria were washed three times with 0.1 M cacodylate buffer and post‐fixed with 1% osmium tetroxide for 1 h. After three washes with water, bacteria were further dehydrated using ethanol gradients (10 min each: 50%, 60%, 70%, 80%, 90%, and 100%). Samples were infiltrated with Epon resin and polymerized at 75°C for 48 h. Ultrathin sections were cut with a diamond knife, picked up on copper grids, and stained with lead citrate. Cell micrographs were examined using a JEM 1011 TEM (JEOL, Tokyo, Japan).

### 4.12 Expression and Purification of LptC

*E. coli* BL21(DE3) cells harboring the pET‐30a (+)‐LptC plasmid were cultured in LB medium supplemented with 50 µg/mL kanamycin at 37°C until the optical density at OD_600_ reached ∼0.5. Protein expression was induced with 0.5 mM isopropyl β‐D‐1‐thiogalactopyranoside (IPTG), followed by incubation at 16°C for 20 h. Cells were harvested by centrifugation (8,000 × g, 20 min, 4°C) and resuspended in lysis buffer. Cell disruption was performed via sonication (160 W) on ice. The lysate was centrifuged (13 000 × g, 20 min, 4°C) to separate soluble and insoluble fractions. Both supernatant and pellet fractions were analyzed by SDS‐PAGE. His‐tagged LptC was purified from both fractions using Ni^2+^‐affinity chromatography according to the manufacturer's protocol for His‐tagged fusion proteins. Eluted fractions containing purified protein were pooled and buffer‐exchanged for downstream applications [53].

### 4.13 Surface Plasmon Resonance (SPR) Analysis

Experiments were performed at 25°C on a BIAcore 1K using CM5 sensor chips, and data were analysed using BIAcore 1K Evaluation software (Cytiva) following the manufacturer's instruction [54]. In brief, a cell on the CM5 sensor chip was activated with a mixture of 200 µM 1‐ethyl‐3‐(3‐dimethylaminopropyl) carbodiimide (EDC) and 50 µM N‐hydroxysuccinimide (NHS) at 10 µL/min for 420 s. A total of 50 µL of protein by mixing with 180 µL of 10 mm sodium acetate solution, pH 5.0, was then immobilized on the surface of the cell at 10 µL/min for 420 s for two repetitive runs. The cell was then blocked with 1 M ethanolamine (10 µL/min for 420 sec). A neighboring aisle that served as a reference was similarly activated and blocked, except that PBS adjusted to pH 5.0 was used for immobilization. Both aisles were then equilibrated with PBS. Molecule stock solution was diluted to a series of concentrations in PBS, and was flowed at 10 µL/min for 150 s in each run. At the end of each flow, cells were regenerated for 5 min with 10 mM glycine‐HCl (pH 2.0) solution at 10 µL/min. Data from the sample cell were collected using Biacore Insight (v. 2.0, Cytiva), and were subtracted by those from the reference cell. Association and dissociation constants were obtained by global fitting of the data to a 1:1 Langmuir binding model using BIAcore 1K Evaluation software (Cytiva, Marlborough, MA, USA).

### 4.14 Resistance Development and Identification

To obtain C4‐induced reduced‐susceptibility mutants, *A. baumannii* ATCC 19606 was serially passaged in cation‐adjusted Mueller–Hinton broth (CAMHB) containing stepwise increasing concentrations of C4. Briefly, overnight cultures were diluted into fresh medium containing subinhibitory concentrations of C4, and cultures exhibiting visible growth were sequentially transferred to medium containing the same or a higher C4 concentration. This procedure was repeated until a stable mutant exhibiting an elevated C4 minimum inhibitory concentration (MIC) was obtained. Genomic DNA was extracted from both the parental strain and the C4‐resistant mutant, and genes encoding components of the Lpt transport system were amplified by PCR using gene‐specific primers and subjected to Sanger sequencing. Sequence alignment between mutant and parental alleles was performed to identify nucleotide substitutions.

### 4.15 Confocal Laser Scanning Microscopy (CLSM)

*A. baumannii* 19606 exposed to C4 (8 µg/mL), colistin (0.03125 µg/mL), or their combination were imaged using CLSM. Briefly, bacterial cultures were inoculated into sterile 6‐well plates containing the drugs. Sterile cell slides were placed in the medium to allow bacteria growth, and incubation was carried out for 24 h at 37°C. After rinsing three times with PBS to remove planktonic cells, bacterial viability was assessed using a bacterial viability kit. After a 15‐min incubation in the dark, the stained discs were examined under a CLSM microscope (Leica TCS SP2, Heidelberg, Germany).

### 4.16 Enzyme‐Linked Immunosorbent Assay (ELISA)

Cytokine levels (IL‐4, IL‐6, IL‐10, IL‐22, TNF‐α, IL‐1β, and IFN‐γ) were quantified using commercial ELISA kits (MLbio, Shanghai, China) according to the manufacturer's instructions. Briefly, 50 µL of serially diluted standards was added to the designated standard wells. For sample wells, 40 µL of sample diluent and 10 µL of sample were combined. Subsequently, 100 µL of horseradish peroxidase (HRP)‐conjugated detection antibody was added to all wells. The plate was sealed and incubated at 37°C for 60 min. Following incubation, the plate was washed five times with wash buffer (30 s per wash) and blotted dry. Chromogen solutions A and B (50 µL each) were then sequentially added to each well, and the plate was incubated at 37°C for 10 min in the dark. The reaction was terminated by adding 50 µL of stop solution. Absorbance was measured at 450 nm using a microplate reader, and cytokine concentrations were determined by interpolating sample optical density (OD) values against the standard curve [55].

### 4.17 Quantitative Real‐Time PCR Analysis

Total RNA was extracted from *A. baumannii* 19606 and RAW264.7 cells using the RNeasy Mini Kit (Vazyme, Nanjing, China) following the manufacturer's instructions. A total of 800 ng purified RNA was reverse transcribed into cDNA using the RT Master Kit (Takara, Shiga, Japan). The PCR reaction mixture was prepared using TB Green Premix Ex Taq (Vazyme). Amplification parameters were as follows: initial denaturation at 95°C for 30 s, followed by 40 cycles of 95°C for 5 s, 60°C for 30 s, and 72°C for 45 s. The 16S rRNA expression level was used as an internal control, and changes in other gene expressions were analyzed using the comparative CT method. Primers are listed in Table S4, synthesized by Youkang Biotech.

### 4.18 Lipidomic Analysis

The lipidomic levels of *A. baumannii* 19606 were analyzed after treatment with C4 (8 µg/mL) in log‐phase bacteria. Lipid extracts (15 µL, 10 mg/mL in methanol) were analyzed using a Waters Acquity UPLC CSH C18 column (2.1 × 150 mm, 1.7 µm) at 55°C with a Thermo Accela 1250 UHPLC coupled to a Thermo Exactive Orbitrap mass spectrometer (negative ESI mode). Separation was performed at 300 µL/min with a linear gradient from 5% to 90% mobile phase B (5 mM ammonium formate in water: acetonitrile:1‐butanol, 0.5:10:90) over 36.5 min. MS data were acquired over m/z 250–2000, and fragmentation spectra were obtained using CID at 80 eV. Data were processed using Thermo Xcalibur software [56].

### 4.19 Gene Knockdown and Overexpression

Plasmid construction was performed by Fenghui Biotechnology (Hunan, China). For CRISPR interference (CRISPRi)‐mediated knockdown of lptC in *A. baumannii* ATCC 19606, a single‐guide RNA (sgRNA) targeting the coding region of lptC was designed and cloned into the CRISPRi vector to generate psgRNA‐*lptC*. This plasmid was introduced into *A. baumannii* 19606 harboring dCas9 by electroporation, yielding strain 19606/psgRNA‐*lptC*. For overexpression, the full‐length *lptC* coding sequence was amplified and cloned into the arabinose‐inducible vector pBAD44 to construct pBAD‐*lptC*, which was then transformed into *A. baumannii* 19606 to generate strain 19606/pBAD‐*lptC*. Transformants were selected on LB agar containing appropriate antibiotics, and *lptC* overexpression was induced with L‐arabinose where indicated.

### 4.20 Gene Knockout

The knockout method followed a previously described protocol with some modifications. Primers used for PCR are listed in Table S3 and were synthesized by Youkang Biotech (Nanjing, China). Due to the chloramphenicol resistance inherent in *A. baumannii* 19606, we used the pRE112 knockout vector with tetracycline resistance. Use PCR to amplify the upstream and downstream fragments of the target gene, and then connect them to a linearized vector through homologous recombination of multiple fragments. Next, the constructed plasmid was transformed into the *E. coli* x7213 competent state by electroporation, and the *A. baumannii* 19606 knockout strain was obtained through homologous substitution.

### 4.21 RNA Sequencing

The transcriptome expression levels of *A. baumannii* 19606 were analyzed after treatment with C4 (8 µg/mL) in log‐phase bacteria. Briefly, three independent biological replicates of *A. baumannii* 19606 cultures were treated for 8 h. After treatment, the bacterial suspension was centrifuged (5,000 × g, 5 min), and the total RNA was extracted and quantified using a Nanodrop spectrophotometer (Thermo Scientific, MA, USA), and sequencing was performed using the Illumina Hiseq 2000 system (Majorbio, Shanghai, China). Differential gene expression was analyzed using the edgeR software. Genes with a false discovery rate (FDR) < 0.05 and |log_2_Fold change| ≥ 1 were considered significantly differentially expressed [57].

### 4.22 Outer Membrane and Cell Membrane Permeability

The fluorescent dyes *N*‐phenyl‐1‐naphthylamine (NPN) was used to evaluate the permeability of the bacterial outer membrane, respectively [58]. Briefly, bacterial suspensions were incubated with final doses of NPN (10 µM) for 30 min, and then C4 alone or in combination with colistin was applied for 1 h. Using an Infinite E Plex Microplate reader (Tecan, Männedorf, Switzerland) with excitation/emission wavelengths of 350 nm/420 nm, the permeability of the outer membrane was then measured.

Cell membrane permeability was measured using propidium iodide (PI) [59]. Briefly, bacterial suspensions were incubated with PI (final concentration, 5 µM) for 30 min, followed by treatment with C4, colistin, or their combination for 1 h. Membrane permeability was measured using an Infinite E Plex microplate reader at excitation/emission wavelengths of 535 nm/615 nm.

### 4.23 Membrane Fluidity Assay

Bacterial cells in the exponential growth phase were harvested by centrifugation, washed twice with PBS, and incubated with 10 µm Laurdan at 37°C for 30 min in the dark. After washing twice with PBS to remove unbound dye, the labeled cells were treated with drugs at 37°C for 1 h. Laurdan fluorescence was measured using an Infinite E Plex Microplate reader (Tecan) at excitation and emission wavelengths of 350 nm and 435/490 nm, respectively. The generalized polarization (GP) value was calculated as GP = (I_435_ − I_490_) / (I_435_ + I_490_), where I_435_ and I_490_ represent the fluorescence intensities at 435 and 490 nm, respectively.

### 4.24 ROS Measurement

ROS levels were measured using 2’,7’‐dichlorodihydrofluorescein diacetate (DCFH‐DA, 10 µM) [60]. The samples were incubated with DCFH‐DA at 37°C for 30 min, washed twice with PBS to reduce extracellular fluorescence, and then treated with C4, colistin, or their combination for 1 h. Fluorescence intensity (λ_excitation_, 488 nm; λ_emission_, 525 nm) was measured using a microplate reader.

### 4.25 Hemolysis Activity

The hemolytic rate of C4 was examined using sterile defibrated sheep erythrocytes in line with a preceding study [61]. Simply speaking, 8% sheep erythrocytes were prepared and incubated with serial‐concentration of C4 peptide (0 to 512 µg/mL) under 37°C for 1 h, sterile PBS serving as blank control and ddH_2_O serving as a positive control. After incubation, the absorbance of the supernatant at 576 nm was detected, and the hemolysis rate was estimated based on the controls.

### 4.26 Ethical Statement and Animal Studies

This study was conducted according to the relevant guidelines of Jiangsu Laboratory Animal Welfare and Ethical of Jiangsu Administrative Committee of Laboratory Animals (SYXK‐2022‐0044). All animal experiments were approved by the Animal Care Committee of Yangzhou University.

### 4.27 Safety Evaluation

In vivo toxicity evaluation model was conducted using a previously published method with slight modifications. 7‐week‐old BALB/c male mice (22 to 25 g) were used in the safety evaluation of C4. These mice were divided into two groups randomly, C4 was 30 mg/kg, and DMSO was intraperitoneally injected as a negative control group. Body weight was recorded during normal feeding for 2 and 14 days, with blood (1 mL) collected from mice by cardiac puncture. Serum biochemical indices include alanine aminotransferase (ALT), aspartate aminotransferase (AST), urea (UREA) and serum creatinine (CREA). Sodium (Na^+^), potassium (K^+^) and chloride (Cl^−^) ion levels in blood were analyzed after 14‐day treatment. Mice were euthanized by cervical dislocation after 14 days post‐inoculation, and liver and kidney were collected for histological pathology slides following standard staining procedures.

### 4.28 Neutropenic Mouse Pneumonia Model

Female ICR mice (*n* = 8 per group) were first rendered neutropenic by cyclophosphamide (two consecutive doses of 150 and 100 mg/kg delivered on 4 and 1 day before infection) [62]. Using a P50‐P200 pipettor, apply 50 µL *A. baumannii* 19606 (1 × 10^8^ CFUs per mouse) suspension to the nostril as the animal inhales, breathing in the bacterial suspension [63]. At 2 h post infection, C4 (5 mg/kg) or colistin (2.5 mg/kg) alone or in combination (5 + 2.5 mg/kg) were given by intraperitoneal injections. At 4 h post infection, mice were euthanized by cervical dislocation. The lungs were aseptically removed, homogenized, serially diluted, and plated on LBA to count bacterial numbers after incubated at 37°C for 24 h.

### 4.29 Neutropenic Mouse Thigh Infection Model

Female ICR mice (*n* = 8 per group) were first rendered neutropenic by cyclophosphamide (two consecutive doses of 150 and 100 mg/kg delivered on 4 and 1 day before infection). Then, 100 µL of *A. baumannii* 19606 bacterial suspensions (1.0 × 10^7^ CFUs per mouse) was injected into the right thighs of each mouse. At 2 h post infection, C4 (5 mg/kg) or colistin (2.5 mg/kg) alone or in combination (5 + 2.5 mg/kg) were given by intraperitoneal injections. At 24 h post infection, mice were euthanized by cervical dislocation. The right thighs were aseptically removed, homogenized, serially diluted, and plated on LBA to count bacterial numbers after incubated at 37°C for 24 h.

### 4.30 Statistical Analyses

Statistical analyses were performed using GraphPad Prism 9.5.0 (Software Inc, San Diego, CA, USA). The data were presented as the mean ± standard deviation (SD). Statistical significance was determined using an unpaired two‐tailed Student's *t*‐test when there were only two groups or by one‐way ANOVA with Dunnett's or Tukey's post‐hoc test if there were more than two groups. Differences between groups were considered significant at *p* < 0.05. (*p < 0.05, **p < 0.01, ***p < 0.001, ****p < 0.0001).

## Author Contributions

Y.L. conceived, designed, and supervised the study. J.L., H.Z., J.C., and S.Z. conducted experiments. J.L., H.Z., and Z.W. analyzed the data. Y.L. and J.L. wrote the manuscript. All authors reviewed, revised, and approved the final manuscript.

## Conflicts of Interest

The authors declare no conflicts of interest.

## Supporting information




**Supporting File**: advs76198‐sup‐0001‐SuppMat.docx.

## Data Availability

All data supporting the findings of this study are available from the corresponding author upon reasonable request.
