# Supplementary material for: Targeting Lipopolysaccharide Transport Induces Membrane Lipid Remodeling and Sensitizes Acinetobacter baumannii to Colistin Treatment
Source: Adv Sci (Weinh). 2026 Jun 19;13(51):e76198. doi: 10.1002/advs.76198 (PMC13336379; doi:10.1002/advs.76198)
Supplement: Supplementary file 1 [file ADVS-13-e76198-s001.docx]

**Supplementary Information**

**Supplementary Figures**

**Supplementary Figure 1. Workflow of structure-based virtual screening.**

Workflow of the structure-based virtual screening strategy, including protein preparation, compound library setup, hierarchical screening using Glide SP and MMGBSA, followed by protein-ligand interaction fingerprint (PLIF) analysis, diversity assessment, and binding mode prediction.

**Supplementary Figure 2. Docking score analysis of virtual screening hits.**

Docking results from the LF1000 and T001 compound libraries were evaluated using glide gScore, binding free energy (dG), and ligand strain energy (LSE) to prioritize hits for further analysis.

**Supplementary Figure 3. Protein–ligand interaction fingerprint (PLIF) and binding mode analysis of candidate compounds.**

(A) PLIF analysis was performed for four predicted binding sites (Site 1 to Site 4). In Site 1, His203 (chain A) and Asn204 (chain B) of the LptB subunit were the most frequently interacting residues. In Site 2, Asn89 and Arg95 of LptF; in Site 3, Asp77 of LptB (chain B) and Tyr353 of LptG; in Site 4, Glu58, Glu249 and Phe33 of LptC were most frequently involved in binding. (B) Representative 2D and 3D binding conformations of hit compounds, with amino acid residues within 4 Å shown.

**Supplementary Figure 4. Expression and purification of LptC protein.**

(A) PCR amplification of the *lptC* gene from *A. baumannii* 19606. (B) Cloning verification of *lptC* into the pET-30 a (+) expression vector. (C) SDS-PAGE analysis of LptC expression in E. coli, showing supernatant (lane 1) and pellet (lane 2) fractions. (D) Purity assessment of the His-tagged LptC protein after affinity chromatography.

**Supplementary Figure 5. *lptC* mutation confer reduced susceptibility to C4.**

(A) MIC analysis of WT and C4-induced mutants with decreased susceptibility. (B) Identification of a nucleotide substitution in *lptC* by Sanger sequencing.

 **Supplementary Figure 6.** **Genetic modulation of *lptC* expression alters C4 susceptibility in *A. baumannii* 19606.**

(A) RT-qPCR validation of *lptC* knockdown and overexpression strains. (B) Effects of *lptC* knockdown and overexpression on C4 susceptibility. Data are shown as mean ± SD. *P* value was determined by one-way ANOVA (A). *****P* < 0.0001.

**Supplementary Figure 7.** **Checkerboard assay of C4 and colistin against colistin-susceptible and -resistant Gram-negative strains.**

The synergistic activity of C4 and colistin against *K. pneumoniae*, *E. coli*, *Salmonella*, and *P. aeruginosa*.

**Supplementary Figure 8.** **CLSM imaging of live/dead bacteria upon C4 and colistin exposure.**

**Supplementary Figure 9. Transcriptomic response of *A. baumannii* to C4 treatment.**

(A) Volcano plot of differentially expressed genes in C4-treated *A. baumannii*. (B) Heatmap of membrane transport related and phospholipid metabolism-related genes. Red indicates downregulation, gray indicates upregulation. (C) RT-qPCR validation of representative genes involved in phospholipid metabolism. (D) RT-qPCR validation of representative genes involved in membrane transport related genes. Data are shown as mean ± SD. *P* value was determined by two-way ANOVA (C, D). **P* < 0.05, ***P* < 0.01, ****P* < 0.001, *****P* < 0.0001. ns, not significant.

**Supplementary Figure 10. Lipidomic profiling of the C4-treated group.**

(A) PCA plot of lipidomic profiles between C4-treated and control samples (n = 6). (B) Volcano plot of differentially regulated lipid metabolites. (C) Heatmap clustering by lipid class revealed notable changes in CL, PE, and PG abundance. (D) Functional lipid enrichment via the LION database. (E) Quantitative comparison of key lipid species, including CL and PE between C4 and control groups. Data are shown as mean ± SD. *P* value was determined by unpaired *t*-test (E). *****P* < 0.0001.

**Supplementary Figure 11. Volcano plots categorized by lipid classes.**

Each subpanel represents a volcano plot of a specific lipid class, displaying all detected lipid species within that category. The x-axis indicates the log_2_ fold change in lipid abundance between the two groups, while the y-axis represents the -log_10_ of the p-value. Lipids with a larger absolute value on the x-axis show greater differences in expression levels, and a higher y-axis value indicates stronger statistical significance. Data points are color-coded: red indicates significantly upregulated lipids, blue denotes significantly downregulated lipids, and gray represents lipids that did not meet the significance threshold.

**Supplementary Figure 12. iPath analysis of metabolic pathway perturbations.**

iPath results highlighted major metabolic pathways affected by C4 treatment, including fatty acid degradation, biosynthesis, and glycerophospholipid metabolism.

**Supplementary Figure 13. MICs analysis of C4 and colistin for the *mlaC* overexpression and complementation strains.**

**Supplementary Figure 14. RT-qPCR validation of genes involved in two-component system, ABC transporters, and lipid metabolism.** Data are shown as mean ± SD. *P* value was determined by two-way ANOVA. *****P* < 0.0001.

**Supplementary Figure 15. Serum biochemistry analysis of mice on Day 2.** Data are shown as mean ± SD. *P* value was determined by t unpaired *t*-test. ns, not significant.

**Supplementary Tables**

**Supplementary Table 1.** **Bacterial strains and plasmids used in this study.**

| Plasmids or strains | Description | Sources |
| --- | --- | --- |
| Plasmids |  |  |
| pRE112 | Genome editing vector, Tet^r^, SacB | Lab stock |
| pRE112-*pgpA-*UP/DOWN | pRE112 with left and right homologous arms for *pgpA* gene | This study |
| pRE112-*pgpB-*UP/DOWN | pRE112 with left and right homologous arms for *pgpB* gene | This study |
| pRE112-*mlaC-*UP/DOWN | pRE112 with left and right homologous arms for *mlaC* gene | This study |
| pRE112-*lolE-*UP/DOWN | pRE112 with left and right homologous arms for *lolE* gene | This study |
| pRE112-*fadE-*UP/DOWN | pRE112 with left and right homologous arms for *fadE* gene | This study |
| pBAD44 | Gene complementation vector, Km^r^ | Lab stock |
| PBAD-*mlaC* | pBAD with *mlaC* gene | This study |
| pET-30a (+) | *E. coli* protein expression vector | Lab stock |
| pET-*lptC* | pET-30a (+) with *lptC* gene | This study |
| psgRNA | Gene knockdown vector, Kan^r^ | Lab stock |
| dCas9 | Gene knockdown vector, Cm^r^ | Lab stock |
| Strains |  |  |
| *A. baumannii* 19606 | ATCC | Lab stock |
| C4-Mutant | C4-induced resistant strain of A. baumannii 19606 | This study |
| *K. pneumoniae* KP-K6 | ATCC | Lab stock |
| *K. pneumoniae* D120 | *mcr* resistant strain | Lab stock |
| *Salmonella* 14028 | ATCC | Lab stock |
| *Salmonella* 091 | *mcr* resistant strain | Lab stock |
| *E. coli* K88 | Enteropathogenic *E. coli* | Lab stock |
| *E. coli* K88*-mcr1* | *mcr* resistant strain | Lab stock |
| *P. aeruginosa* PA01 | ATCC | Lab stock |
| *P. aeruginosa* 8-1 | Clinically highly resistant bacterial strain | Lab stock |
| *E. coli* DH5α | F^-^, φ 80d*lacZ* ΔM15, Δ(*lacZYA* -*argF*)U169, *deoR*, *recA1*, *endA1*, *hsdR17*(*rK*^-^, *mK^+^*), *phoA*, *supE44*, *λ^-^*, *thi -1*, *gyrA96* , *relA1* | Lab stock |
| *E. coli* DH5α λpir | F- φ80lacZΔM15 Δ (lacZYA-argF) LAMpir U169 endA1 recA1 hsdR17(r_k_^-^, m_k_^+^) supE44λ- thi -1 gyrA96 relA1 phoA | Lab stock |
| BL21 (DE3) | *E. coli* proteinexpression strain | Lab stock |
| *E. coli* x7213 | *Thi21 thr21 leuB6f huA21 lacY1glnV44△asdA4 rexA1 RP422Tc:: Mu [*λpir*] Kmr* | Lab stock |
| pET-30a (+)-LptC | *E. coli* DH5α with LptC gene sequence | This study |
| BL21 (DE3)- pET-30a (+)-LptC | BL21 (DE3) with pET-30a (+)-LptC | This study |
| *E. coli* x7213-pRE112-*pgpA-*UP/DOWN | E. coli X7213 with recombinant plasmid pRE112-*pgpA-*UP/DOWN, Tet^r^, SacB | This study |
| *E. coli* x7213-pRE112- *pgpB-*UP/DOWN | E. coli X7213 with recombinant plasmid pRE112- *pgpB-*UP/DOWN, Tet^r^, SacB | This study |
| *E. coli* x7213-pRE112- *mlaC-*UP/DOWN | E. coli X7213 with recombinant plasmid pRE112- *mlaC-*UP/DOWN, Tet^r^, SacB | This study |
| *E. coli* x7213-pRE112- *lolE-*UP/DOWN | E. coli X7213 with recombinant plasmid pRE112- *lolE-*UP/DOWN, Tet^r^, SacB | This study |
| *E. coli* x7213-pRE112- *fadE-*UP/DOWN | E. coli X7213 with recombinant plasmid pRE112- *fadE-*UP/DOWN, Tet^r^, SacB | This study |
| *A. baumannii* *-*Δ*pgpA* | Single deletion mutant in *pgpA* in the background of *A. baumannii* 19606 | This study |
| *A. baumannii* *-*Δ*pgpB* | Single deletion mutant in *pgpB* in the background of *A. baumannii* 19606 | This study |
| *A. baumannii* *-*Δ*mlaC* | Single deletion mutant in *mlaC* in the background of *A. baumannii* 19606 | This study |
| *A. baumannii* *-*Δ*lolE* | Single deletion mutant in *lolE* in the background of *A. baumannii* 19606 | This study |
| *A. baumannii* *-*Δ*fadE* | Single deletion mutant in *fadE* in the background of *A. baumannii* 19606 | This study |
| 19606/pBAD | 19606 containing pBAD empty vector | This study |
| 19606/pBAD-*lptC* | overexpressing strain of *lptC* gene in 19606 | This study |
| 19606/pBAD-*mlaC* | overexpressing strain of *mlaC* gene in 19606 | This study |
| Δ*mlaC*/pBAD-*mlaC* | *mlaC* gene complementation strain of Δ*mlaC* | This study |
| 19606/psgRNA | 19606 containing psgRNA empty vector | This study |
| 19606/psgRNA-*lptC* | *lptC* gene knockdown strain of 19606 | This study |

**Supplementary Table 2. Information of selected high affinity compounds and antimicrobial activity against *A. baumannii* 19606 (MIC, μg/mL).**

| Number | Compounds | Site | Formula | MolWt | Purity (%) | Solvent | MIC |
| --- | --- | --- | --- | --- | --- | --- | --- |
| A2 | HA130 | 2 | C24H19BFNO5S | 463.29 | ≥95 | DMSO | 512 |
| A3 | Suramin Sodium Salt | 2 | C51H34N6Na6O23S6 | 1429.15 | 99.76 | DMSO | 512 |
| A4 | Linarin | 2 | C28H32O14 | 592.55 | 98.27 | DMSO | 256 |
| A5 | Pinoresinol diglucoside | 3 | C32H42O16 | 682.67 | 99.90 | DMSO | 512 |
| A6 | L755507 | 2 | C30H40N4O6S | 584.73 | 99.75 | DMSO | 512 |
| A7 | Ertugliflozin | 2 | C22H25ClO7 | 436.88 | 99.96 | DMSO | 512 |
| A8 | Argipressin acetate (113-79-1(free base)) | 4 | C48H69N15O14S2 | 1144.28 | 98.87 | DMSO | 512 |
| A9 | Imidazole ketone erastin | 3 | C35H35ClN6O5 | 655.14 | 99.89 | DMSO | 512 |
| A10 | Lanreotide acetate | 4 | C54H69N11O10S2 | 1096.33 | >99.99 | DMSO | 512 |
| A11 | Adenosine amine congener | 3 | C28H32N8O6 | 576.6 | 97.86 | DMSO | 512 |
| B2 | Magnesium Lithospermate B | 2 | C36H28MgO16 | 740.9 | 97.5 | DMSO | 512 |
| B3 | ATN-161 trifluoroacetate salt | 2 | C25H36F3N9O10S | 711.67 | 99.92 | DMSO | 512 |
| B4 | Sincalide | 3 | C49H62N10O16S3 | 1143.27 | 98.46 | DMSO | 512 |
| B5 | PROTAC BRAF-V600E degrader-1 | 3 | C48H54F2N10O10S | 1001.07 | 99.43 | DMSO | 512 |
| B6 | ARV-771 | 4 | C49H60ClN9O7S2 | 986.64 | 99.69 | DMSO | 512 |
| B7 | BMS-1001 hydrochloride | 2 | C35H35ClN2O7 | 631.11 | 97.36 | DMSO | 512 |
| B8 | FSLLRY-NH2 TFA (245329-02-6 free base) | 4 | C41H61F3N10O10 | 910.99 | 99.32 | DMSO | 512 |
| B9 | NSC781406 | 2 | C29H27F2N5O5S2 | 627.68 | 99.58 | DMSO | 512 |
| B10 | FGFR1/DDR2 inhibitor 1 | 2 | C28H22F3N5O | 501.5 | 99.43 | DMSO | 256 |
| B11 | Procyanidin A1 | 2 | C30H24O12 | 576.5 | 99.52 | DMSO | 512 |
| C2 | Abz-FR-K(Dnp)-P-OH acetate (500799-61-1 free base) | 3 | C41H53N11O12 | 891.95 | >99.99 | DMSO | 256 |
| C3 | Tetrahydrodehydrodiconiferyl alcohol | 2 | C20H26O6 | 362.42 | 99.54 | DMSO | 512 |
| C4 | BIM 23042 Acetate | 4 | C65H77N11O11S2 | 1252.5 | 98.63 | DMSO | 64 |
| C5 | Enterobactin | 1 | C30H27N3O15 | 669.55 | 96.73 | DMSO | 512 |
| C6 | EGFR-IN-8 | 3 | C32H23ClF3N7O4 | 662.02 | 99.51 | DMSO | 512 |
| C7 | FMoc-Val-Cit-PAB-PNP | 4 | C40H42N6O10 | 766.8 | 98.05 | DMSO | 512 |
| C8 | Forsythiaside A | 1 | C29H36O15 | 624.59 | 99.95 | DMSO | 512 |
| C9 | Plantainoside D | 2 | C29H36O16 | 640.59 | 99.97 | DMSO | 512 |
| C10 | HS024 | 4 | C58H79N19O10S2 | 1266.5 | >99.99 | DMSO | 64 |
| C11 | Dasatinib N-oxide | 2 | C22H26ClN7O3S | 504.01 | 99.94 | DMSO | 512 |
| D2 | SMCC-DM1 | 4 | C51H66ClN5O16S | 1072.61 | 97.07 | DMSO | 512 |
| D3 | Boc-Val-Cit-PAB-PNP | 2 | C30H40N6O10 | 644.67 | 98.21 | DMSO | 256 |
| D4 | Compound F0806-0303(SC) | 3 | C32H22N6O2S | 554.63 | >90 | DMSO | 512 |
| D5 | Compound F0816-0342(SC) | 1 | C40H36N6O9S4 | 873.009 | >90 | DMSO | 512 |
| D6 | Compound F6412-1681(SC) | 2 | C18H16F3N3O4 | 395.338 | >90 | DMSO | 512 |
| D7 | Compound F6041-0243(SC) | 2 | C22H18N4O2S2 | 434.53 | >90 | DMSO | 512 |
| D8 | Compound F2728-0881(SC) | 4 | C30H28Cl2N4O6S4 | 739.72 | >90 | DMSO | 512 |
| D9 | Compound F1507-0084(SC) | 2 | C29H31NO10 | 553.564 | >90 | DMSO | 512 |
| D10 | Compound F6507-3756(SC) | 2 | C18H19NO5 | 329.352 | >90 | DMSO | 512 |
| D11 | Compound F1507-0100(SC) | 2 | C27H29NO11 | 543.525 | >90 | DMSO | 512 |
| E2 | Compound F0721-0722(SC) | 2 | C28H30N6O4S2 | 578.71 | >90 | DMSO | 512 |
| E3 | Compound F0398-0929(SC) | 2 | C26H18O4 | 394.426 | >90 | DMSO | 512 |
| E4 | Compound F2716-0252(SC) | 2 | C19H16N2O3S | 352.41 | >90 | DMSO | 256 |
| E5 | Compound F2478-0045(SC) | 2 | C19H15ClN2O3S | 386.85 | >90 | DMSO | 512 |
| E6 | Compound F6351-0010(SC) | 2 | C18H17N9O2 | 391.395 | >90 | DMSO | 512 |
| E7 | Compound F5981-0239(SC) | 2 | C22H24N4O4 | 408.458 | >90 | DMSO | 256 |
| E8 | Compound F0806-0342(SC) | 3 | C40H28N6O3 | 640.703 | >90 | DMSO | 512 |
| E9 | Compound F0207-0386(SC) | 1 | C23H20N4O7S3 | 560.61 | >90 | DMSO | 512 |
| E10 | Compound F3394-0860(SC) | 2 | C24H25N3O5S2 | 499.6 | >90 | DMSO | 512 |
| E11 | Compound F0375-0327(SC) | 1 | C46H34N4O2 | 674.7878 | >90 | DMSO | 512 |
| F2 | Compound F0336-0063(SC) | 2 | C21H20N2O3S | 380.46 | >90 | DMSO | 512 |
| F3 | NADP disodium salt | 3 | C21H26N7Na2O17P3 | 787.37 | 99.56 | H_2_O | 512 |
| F4 | Diquafosol tetrasodium | 3 | C18H22N4Na4O23P4 | 878.23 | 99.97 | H_2_O | 512 |
| F5 | Compound F6036-1438(SC) | 4 | C24H25N3O6 | 451.47198 | >90 | DMSO | 512 |
| F6 | Compound F6246-0587(SC) | 4 | C25H25N3O2S | 431.54999 | >90 | DMSO | 512 |
| F7 | Compound F6246-0079(SC) | 4 | C27H26FN3O2 | 443.513 | >90 | DMSO | 512 |
| F8 | Compound F6497-2103(SC) | 4 | C20H22N4O2S | 382.479 | >90 | DMSO | 512 |
| F9 | Afoxolaner | 4 | C26H17ClF9N3O3 | 625.87 | >90 | DMSO | 512 |
| F10 | LY2510924 acetate (1088715-84-7 free base) | 4 | C64H91N13O13 | 1250.49 | >90 | DMSO | 512 |
| F11 | Angiotensin amide acetate | 4 | C51H73N14O13 | 1090.21 | >90 | DMSO | 512 |
| G2 | Brilliant blue G-250 | 4 | C47H48N3NaO7S2 | 854.02002 | >90 | DMSO | >128 |
| G3 | Edotreotide | 4 | C65H92N14O18S2 | 1421.64 | >90 | DMSO | >128 |
| G4 | Felypressin acetate | 4 | C48H69N13O13S2 | 1100.27 | >90 | DMSO | >128 |
| G5 | Batefenterol | 4 | C40H42ClN5O7 | 740.23999 | >90 | DMSO | >128 |
| G6 | TRAP-6 | 4 | C34H56N10O9 | 748.87 | >90 | DMSO | >128 |
| G7 | Terlipressin Acetate | 4 | C56H82N16O19S2 | 1347.5 | >90 | DMSO | >128 |
| G8 | Endomorphin 1 | 4 | C34H38N6O5 | 610.66998 | >90 | DMSO | >128 |

**Supplementary Table 3. Primers used in this study.**

| **Name** | **Sequence (5’-3’)** | **Description** |
| --- | --- | --- |
| pET-F | TAATACGACTCACTATAGGG | pET-30a (+) identifying primers |
| pET-R | GCTAGTTATTGCTCAGCGG |  |
| pET-LptC-F | CGGAATTCGCCACCATGAGTGGCAAGGCAAAAAAACTGG | Amplification of LptC fragments |
| pET-LptC-R | CCCTCGAGTTAATGATGATGATGATGATGAGACTTTGGTGCATACTTTCCTCGAA |  |
| V-LptC-F | ACGACGACGACAAGGCCATGGCT | confirm the connection of fragment to the vector |
| V-LptC-R | ATGCTAGTTATTGCTCAGCGGT |  |
| PRE-F | CATAGCCCCACTGTTCGTCCAT | pRE112 identifying primers |
| PRE-R | ATCCAAGAACAACCATCCTCCT |  |
| *pgpA*-UP-F | TCGATATCGCATGCGGTACCACTGTTGTAATCAATTT | amplification of ~500bp *pgpA* upstream for gene deletion |
| *pgpA*-UP-R | GACATGAATAACTCGAATGATGAATTTGCAATGGGTAAT |  |
| *pgpA*-DOWN-F | CAGATTACCCATTGCAAATTCATCATTCGAGTTATTCAT | amplification of ~500bp *pgpA* downstream for gene deletion |
| *pgpA*-DOWN-R | ATCCCAAGCTTCTTCTAGATTGCTGACACAGTAAATACCT |  |
| *pgpB*-UP-F | CGATATCGCATGCGGTACCGGCGTAATGTTAGGAAGTAT | amplification of ~500bp *pgpB* upstream for gene deletion |
| *pgpB*-UP-R | GTTCCACCTCGTTTAGATAAAATAAACACACCTTTGCTTA |  |
| *pgpB*-DOWN-F | ATGAATAAGCAAAGGTGTGTTTATTTTATCTAAACGAGGT | amplification of ~500bp *pgpB* downstream for gene deletion |
| *pgpB*-DOWN-R | TCCCAAGCTTCTTCTAGAATAGGAGAGCGAATTGT |  |
| *mlaC*-UP-F | TCGATATCGCATGCGGTACCTTAAAGCCTCGTGCTAAAGT | amplification of ~500bp *mlaC* upstream for gene deletion |
| *mlaC*-UP-R | GATACTGAACCACTCTTCACCTACCTGGAACCACTCCTT |  |
| *mlaC*-DOWN-F | TATTTGAAGGAGTGGTTCCAGGTAGGTGAAGAGTGGTTCA | amplification of ~500bp *mlaC* downstream for gene deletion |
| *mlaC*-DOWN-R | TCCCAAGCTTCTTCTAGAAGTATGTTGGCACCACCTATGT |  |
| *lolE*-UP-F | GATATCGCATGCGGTACCGACTGGTTCGATCTTGGTTTCT | amplification of ~500bp *lolE* upstream for gene deletion |
| *lolE*-UP-R | CTTTTGCTTCTAAAACGACTTCACACTTCTTATTTAGCCAT |  |
| *lolE*-DOWN-F | GCTAAATAAGAAGTGTGAAGTCGTTTTAGAAGCAAAAGAT | amplification of ~500bp *lolE* downstream for gene deletion |
| *lolE*-DOWN-R | CCAAGCTTCTTCTAGAAGGTTACCCGTAGGCTCATCAGCT |  |
| *fadE*-UP-F | TATCGCATGCGGTACCTCGACTATGTCACTATTGGTCAAT | amplification of ~500bp *fadE* upstream for gene deletion |
| *fadE*-UP-R | TGAATTGGTGGAGGCACCAGACTTGCTCCTTGCTAGAT |  |
| *fadE*-DOWN-F | TAGCAAGGAGCAAGTCTGGTGCCTCCACCAATTCACTATT | amplification of ~500bp *fadE* downstream for gene deletion |
| *fadE*-DOWN-R | TCCCAAGCTTCTTCTAGATGTCGTAATTGGTGACCGTAAT |  |
| VER-*pgpA*-F | TGAGTGGCTTGAAGGATTTAGT | verify the knockout of *pgpA* gene knockout |
| VER-*pgpA*-R | CTAAACCATCAGCAACAGCCAT |  |
| VER-*pgpB*-F | ACTTATTTGGACAAGAACGCCT | verify the knockout of *pgpB* gene knockout |
| VER-*pgpB*-R | TTACTGAAGAACGACCAGATTG |  |
| VER- *mlaC*-F | GAAAGTGAGTGGTTTAGTGGGC | verify the knockout of *mlaC* gene knockout |
| VER- *mlaC*-R | TTGCTTGATACAACGTCAGGAT |  |
| VER- *lolE*-F | TGTCTTAATGTTGGTCAGCGTT | verify the knockout of *lolE* gene knockout |
| VER- *lolE*-R | GATCGGTAAGCAACTCGAAAAT |  |
| VER-*fadE*-F | CCAGGTTCAGATTATCAGCACT | verify the knockout of *fadE* gene knockout |
| VER-*fadE*-R | GCAGCAGTTTTTGAGAATCAAC |  |
| pBAD-*mlaC*-F | TGGGCTAGCAGGAGGAATTCGTGAATACGTTGTTTAAAC | amplification of *mlaC* gene for overexpression and complementation |
| pBAD-*mlaC*-R | CCGCCAAAACAGCCAAGCTTTTATTTTTGTTTATTCTGA |  |
| pBAD-*lptC*-F | tgggctagcaggaggaattcatggataccagagttttat | amplification of *lptC* gene for overexpression and complementation |
| pBAD-*lptC*-R | ccgccaaaacagccaagcttttaagactttggtgcatac |  |
| pBAD-VER-F | TAATCACGGCAGAAAAGTCCAC | confirm the connection of fragment to the vector |
| pBAD-VER-R | TTCTGCGTTCTGATTTAATCTG |  |
| KD-*lptC*-F1 | TGAAACTCAATGGTTCCTTGgttttagagctagaaatagc | amplification of l*ptC* gene’s sgRNA1 for knockdown |
| KD-*lptC*-R1 | CAAGGAACCATTGAGTTTCAactagtattatacctaggac |  |
| KD-*lptC*-F2 | TTGACTCAAACTGATGACAAgttttagagctagaaatagc | amplification of *lptC* gene’s sgRNA2 for knockdown |
| KD-*lptC*-R2 | TTGTCATCAGTTTGAGTCAAactagtattatacctaggac |  |
| KD-*lptC*-F3 | GGTTGTGTTATCAGGTGATGgttttagagctagaaatagc | amplification of *lptC* gene’s sgRNA3 for knockdown |
| KD-*lptC*-R3 | CATCACCTGATAACACAACCactagtattatacctaggac |  |
| KD-VER-F | tgctaggaggtgactgaagta | confirm the connection of fragment to the vector |
| KD-VER-R | gtaccatgggatcctgattacgatc |  |

**Supplementary Table 4. RT-PCR primers used in this study.**

| **Name** | **Sequence (5’-3’)** |
| --- | --- |
| *pgpA*-F | CCTTGGCCTATACGCGTCAT |
| *pgpA*-R | AAAGAGCAGCCCATACACCG |
| *paaF*-F | TGCAGTGTTTGTGCGAGTTG |
| *paaF*-R | AGCTTCGGGCGAATATCGAG |
| *fadE*-F | CCCTGCTGTAATGACTGCCA |
| *fadE*-R | GCCCAAGTTGAATTGCACGT |
| *purK*-F | AAAATTGAAGGCGTGGCGTC |
| *purK*-R | CCGCGTTTGGTTGGCTTTAA |
| *dhaT*-F | GGTTTAACCGCTGCAACAGG |
| *dhaT*-R | AGCATCACGAGCTTCGATGT |
| *ssuA*-F | CCTACTCGTTCAGGCCGTAC |
| *ssuA*-R | TCCTTTTTGGAATGCAGCGC |
| *mlaC*-F | ACAAGGCGACAGCTGGTAAA |
| *mlaC*-R | CCAACTGGAACGACACAGGA |
| *sbp*-F | CGCAAGAGTTTGTTCGCCAA |
| *sbp*-R | ATACGTCACCAATTCCGCGT |
| *lolE*-F | CCATCTCCAGCTGGTGTTGT |
| *lolE*-R | CCTTGAGCGCCATCAGGTAA |
| *vgrG*-F | CGCATCCATGAAGCAGAACG |
| *vgrG*-R | GCTACTTTGCAACTGGGCAC |
| *hcp*-F | CCACACTGCTGAACGTGTTG |
| *hcp*-R | CGTTTATCGCCATTTGCACG |
| *tauB*-F | CCGTCAGGGCGAGTCTATTC |
| *tauB*-R | GTAACGCCTCCTTACCGCTT |
| *mmgC*-F | ATGATGGGTGGAAACGGCAT |
| *mmgC*-R | CAATGCCTGTTTGTGCACGA |
| *rstA*-F | ACCGATGGTAACCGTGCAAT |
| *rstA*-R | TCAGTACGCGCAGTCAACAT |
| *wza*-F | TGCGTGATCAAGGCATGACT |
| *wza*-R | GCCGAAATCACCGAGACTCA |
| *lptC*-F | GCTGCTGTGAGTGGTGGTTA |
| *lptC*-R | CCCTGCGCCGAATAAGTCAT |
| AB16S-F | GACGTACTCGCAGAATAAGC |
| AB16S-R | TTAGTCTTGCGACCGTACTC |
